# Effects of Dietary *Periplaneta americana* Residue Supplementation on Porcine Ovaries and Screening Analysis of Functional Small Peptides

**DOI:** 10.3390/ani16182958

**Published:** 2026-09-20

**Authors:** Chenglong Pan, You Tan, Yujun Wu, Zilin Li, Rong Jiang, Linjie Xu, Birong Zhang, Anran Xu, Ran Pu, Junjun Wang, Shiyan Sui

**Affiliations:** 1 College of Public Health, Dali University, Dali 671000, China; 2 College of Animal Science and Technology, China Agricultural University, Beijing 100083, China; 3 Yunnan Provincial Key Laboratory of Entomological Biopharmaceutical R&D, Dali 671000, China

**Keywords:** *Periplaneta americana* residue, pigs, ovarian function, multi-omics, bioactive peptides

## Abstract

Background: *Periplaneta americana* residue (PAR) has potential as a sustainable insect-derived feed ingredient. This study evaluated the effects of 3% dietary PAR on growth performance and ovarian function in finishing pigs and screened potential bioactive peptides. Methods: Thirty finishing pigs were assigned to a control diet or a diet containing 3% PAR. Growth traits, serum oxidative and inflammatory indicators, and ovarian histomorphology were assessed in vivo. Multi-omics profiling was integrated with quantitative real-time PCR, Western blotting, and immunofluorescence assays. The properties of selected peptides and their potential interactions with candidate proteins were assessed separately using in silico prediction, molecular docking, and molecular dynamics simulations. Results: PAR supplementation increased average daily gain and the ovary-to-body weight ratio and decreased serum malondialdehyde levels and follicular atresia, without significantly affecting feed intake, feed conversion ratio, or inflammatory cytokines. Multi-omics profiling associated PAR supplementation with changes in ovarian metabolism, antioxidant defense, immune regulation, and phosphorylation-related signaling. MGST2 and PPM1K were identified as candidate proteins, and their expression patterns were supported experimentally. Serum peptidomics identified GVSEIQQ as having higher abundance in the PAR group than in the control group, whereas GVEEHETL and PGIPT were detected only in the PAR group. In silico analyses predicted that these peptides were non-toxic, non-allergenic, and potentially antioxidant; these predictions require experimental validation. Molecular docking and molecular dynamics simulations suggested potential interactions of GVSEIQQ with MGST2 and GVEEHETL with PPM1K. However, these computational findings do not establish direct peptide–protein binding or functional effects in vivo. Conclusions: PAR shows potential as an alternative feed ingredient that may partially replace soybean meal while supporting growth and ovarian condition in finishing pigs. Further biochemical and in vivo studies are required to confirm the biological functions and mechanisms of the identified peptides.

## Simple Summary

*Periplaneta americana* residue (PAR) is a by-product of medicinal processing that may be used as an alternative feed ingredient. This study examined the effects of including 3% PAR in the diets of finishing pigs. Pigs receiving PAR had a higher average daily gain and relative ovary weight, lower serum malondialdehyde levels, and fewer atretic follicles than control pigs. Feed intake, feed conversion, and inflammatory indicators were not significantly affected. Laboratory analyses identified changes related to ovarian metabolism and antioxidant defense, along with three candidate small peptides. Computer-based analyses predicted that these peptides may have antioxidant properties and interact with selected proteins. These interactions were predictions only and were not confirmed in the animals. The findings suggest that PAR has the potential to partially replace soybean meal while supporting pig growth and ovarian condition. Further studies are needed to verify the biological roles of the identified peptides and evaluate the long-term and economic benefits of using PAR in pig diets.

## 1. Introduction

Pork is one of the most widely consumed meats worldwide and represents an important source of high-quality dietary protein and various essential micronutrients for humans [1]. With the continuous growth in global meat demand, reproductive efficiency and health status in pigs have become central concerns in animal husbandry, as they are directly associated with production performance and economic returns. Ovarian development is a pivotal determinant of sow reproductive performance, and both the quantity and quality of follicles directly influence oocyte fertilization capacity and eventual reproductive success [2,3]. Under modern intensive farming systems, the nutritional composition of feed exerts profound effects on porcine growth, development, and reproductive performance. Compared with conventional feed ingredients, insect protein, as an emerging protein feed resource, is characterized by a relatively high protein content, a well-balanced amino acid profile, and an abundance of potentially bioactive components. It is therefore regarded as a promising candidate for replacing traditional protein sources in pig diets [4,5]. These bioactive constituents may play critical regulatory roles in ovarian function and follicular development in pigs. Nevertheless, studies investigating the effects of insect protein on reproductive performance in livestock and poultry remain relatively limited.

*Periplaneta americana* L. (PA), a traditional medicinal insect, has attracted considerable attention owing to its broad pharmacological activities. Previous studies have demonstrated that PA extracts exert significant effects in improving osteoporosis and enhancing immune function, and several clinical preparations, such as Kangfuxin Liquid and Xinmailong Injection, have been successfully developed from PA-derived ingredients [6,7,8]. It has been reported that small peptides derived from PA extracts can markedly alleviate H_2_O_2_-induced oxidative stress and apoptosis in porcine ovarian granulosa cells [9]. Additional studies have shown that PA-derived small peptides can inhibit apoptosis in KGN cells [10,11]. *Periplaneta americana* residue (PAR) is the residual material obtained after the bodies of *P. americana* are soaked in 80–95% ethanol for 24 h. Despite undergoing this extraction process, PAR retains substantial amounts of protein and certain bioactive compounds, indicating its potential as an alternative feed resource [12]. In a previous study conducted by our research group, PAR was found to contain up to 68% crude protein and 17 amino acids, including seven essential amino acids. Its crude protein content was comparable to or even higher than that of the Peruvian fish meal. Previous studies have shown that dietary PAR supplementation at appropriate levels can improve growth performance and enhance immune function in Sanhuang chickens, Nile tilapia, and other animal species [13,14]. Moreover, the active compounds retained in the medicinal residues can accelerate wound healing in diabetic rats by promoting wound closure, collagen deposition, M2 macrophage polarization, and angiogenesis [15]. However, the effects of PAR as a feed additive on sow ovaries and the functional small peptides responsible for its biological activity have not yet been reported.

With the rapid development of high-throughput sequencing, mass spectrometry, and bioinformatics, integrated multi-omics analysis has become an important strategy for elucidating how complex nutritional interventions in feed regulate physiological functions. Feedomics can integrate multiple omics technologies to reveal the relationships between nutritional factors, animal production performance, and health status at different molecular levels, thereby providing new insights into the nutritional effects of feed and their underlying molecular mechanisms [16]. Omics approaches, including transcriptomics, proteomics, and metabolomics, have been widely applied in animal nutrition research and can be used to decipher the effects of feed or feed-derived bioactive components on animal metabolism, health, and production traits [17]. Peptidomics, as an important technique in the study of bioactive peptides from food and feed, enables the characterization of peptide fragments generated during protein processing, storage, and digestion and facilitates the identification of food-derived bioactive peptides and peptide biomarkers [18]. After entering the organism, active components in feed, such as proteins, peptides, and polysaccharides, may influence animal growth, development, and reproductive function by regulating gene expression, protein function, metabolic pathways, immune homeostasis, and endocrine signaling. The combined application of transcriptomics, proteomics, metabolomics, and peptidomics can systematically reveal the biological effects of feed intervention from the perspectives of gene regulation, protein execution, metabolic response, and bioactive peptide variation [19]. The HDOCK server can be used for docking prediction of protein–peptide complexes. Based on a hybrid strategy combining template-based modeling and ab initio docking, HDOCK enables the prediction of binding conformations and interaction patterns between candidate peptides and target proteins [20,21]. GROMACS is a widely used molecular dynamics simulation package that can further evaluate the conformational stability, residue flexibility, hydrogen bond dynamics, solvent accessibility, and free energy distribution of protein–peptide complexes in a dynamic environment [22].

In the present study, PAR was supplemented in sow diets, and integrated multi-omics approaches were employed to systematically identify key differentially expressed genes, differentially abundant proteins, differential metabolites, and potential bioactive peptides in ovarian tissues following PAR intervention. Furthermore, molecular docking and molecular dynamics simulations were conducted to analyze the interaction patterns between candidate bioactive peptides and target proteins associated with ovarian development. Through integrated multi-omics analyses, this study aims to elucidate the effects of PAR on ovarian function and follicular development in sows, identify potentially bioactive peptides, and clarify the underlying molecular mechanisms and key regulatory targets involved in porcine reproduction. The findings will provide a scientific basis for evaluating PAR as a novel insect-derived protein feed ingredient and for improving the nutritional regulation of sow reproductive performance. Moreover, this study may promote the high-value and feed-oriented utilization of *Periplaneta americana* medicinal residues, support the development of alternative protein sources, reduce dependence on soybean meal, and lower pig production costs.

## 2. Materials and Methods

### 2.1. Feed Composition

The experimental diets consisted of a control diet based on a corn–soybean meal formulation and a PAR diet, which was prepared by supplementing the control diet with 3% PAR powder. The two diets were formulated to be nutritionally comparable, with crude protein content of approximately 13.5% and digestible energy of approximately 3400 kcal/kg. The contents of key nutrients, including lysine, methionine, and digestible phosphorus, were similar between the two diets, as shown in Table 1.

### 2.2. Biosafety Evaluation of Dietary PAR in Mice

All experimental procedures were approved by the Laboratory Animal Welfare Ethics Committee of Dali University (approval No. 2025PZ0040). Thirty-six healthy male C57BL/6 mice were randomly assigned to six groups, with six mice per group: a control group and five PAR treatment groups receiving diets containing 0.5%, 1%, 5%, 10% and 20% PAR. Mice in the control group were fed a standard maintenance diet, whereas mice in the treatment groups were fed maintenance diets supplemented with the corresponding levels of PAR for 28 days. At the end of the feeding period, the mice were euthanized, and blood and major organs, including the heart, liver, spleen, lung, kidney, testis, and colon, were collected. Blood samples were allowed to stand for 1 h and then centrifuged at 3000 rpm for 15 min. The serum was collected and stored at −80 °C for biochemical analysis. Serum uric acid (UA), alkaline phosphatase (ALP), creatinine (CRE), aspartate aminotransferase (AST), and alanine aminotransferase (ALT) were measured. The collected organs were fixed, sectioned, and subjected to hematoxylin and eosin staining for histopathological examination.

### 2.3. Animal Management and Sample Preparation

This study was approved by the Animal Ethics Committee of Dali University (approval No. 2024-P2-143), and all experimental procedures were conducted in accordance with the institutional guidelines for animal research. A total of 30 sows were individually housed in separate pens under standard environmental conditions, with ad libitum access to feed and water. Following acclimatization, the animals were randomly assigned to the control group (*n* = 15) or the PAR group (*n* = 15), receiving a basal diet or a basal diet supplemented with PAR, respectively. The two diets were formulated to provide comparable nutrient levels, and the feeding trial began simultaneously in both groups. Each individually housed sow was considered an independent experimental unit and biological replicate. After 28 days of feeding, five sows with similar body weights were randomly selected from each group for slaughter and sample collection (*n* = 5 independent biological replicates per group). The animals were electrically stunned and exsanguinated, followed by routine carcass processing and evisceration in accordance with the relevant Chinese guidelines and the regulations of the Animal Ethics Committee of Dali University. Blood samples were collected and processed to obtain serum. Ovarian tissues were collected immediately after slaughter, snap-frozen in liquid nitrogen, and stored at −80 °C until further analysis. Samples from individual animals were processed and analyzed separately without pooling. Because sample sizes varied among subsequent assays, the exact number of biological replicates used for each analysis is specified in the corresponding Methods subsection and figure or table legend. For the multi-omics analyses, the same three sows from each group were used across all analytical platforms, including untargeted metabolomics, amino acid metabolomics, transcriptomics, proteomics, phosphoproteomics, and serum peptidomics. Accordingly, serum samples used for peptidomic analysis were obtained from the same animals whose ovarian tissues were used for the other omics analyses. This consistent sample allocation facilitated cross-platform comparisons and integrative multi-omics analysis.

### 2.4. Ovarian Hematoxylin and Eosin Staining and Follicle Counting

After electrical stunning and slaughter, the ovaries were excised from the five sows selected for sample collection in each group. Among these five sows, three were randomly selected for ovarian histological examination and follicle counting, and each sow was regarded as one independent biological replicate. Five sections were prepared from different levels of each ovary and subjected to hematoxylin and eosin (H&E) staining. The stained sections were independently examined by two blinded observers. The numbers of different follicle types, including primordial follicles, Graafian follicles, also referred to as preovulatory follicles, and atretic follicles, were counted under a light microscope. Finally, the total number of follicles at each developmental stage and the number of atretic follicles were calculated.

### 2.5. Growth Performance and Serum Biochemical Index Detection

During the experimental period, body weight and feed intake were recorded individually for each sow. Average daily gain (ADG), average daily feed intake (ADFI), and feed conversion ratio (FCR) were calculated separately for each animal. Therefore, each sow was regarded as an independent experimental unit and biological replicate for growth-performance analysis (*n* = 15 per group). For serum biochemical analysis, blood samples were collected from the five sows slaughtered for sample collection in each group, as described in Section 2.3. Thus, five independent biological replicates were included in the CO group and five in the PAR group (*n* = 5 per group). Serum was separated by centrifugation, and samples from individual sows were analyzed separately without pooling. Commercial assay kits were used to determine the serum levels of malondialdehyde (MDA), total antioxidant capacity (T-AOC), total superoxide dismutase (T-SOD), glutathione peroxidase (GSH-Px), catalase (CAT), tumor necrosis factor-α (TNF-α), interleukin-1β (IL-1β), and interleukin-6 (IL-6).

### 2.6. Untargeted Metabolomics Analysis

Metabolomics analysis was performed by Shanghai Applied Protein Technology Co., Ltd./APTBIO (Shanghai, China). Samples were thawed at 4 °C, and pre-cooled methanol/acetonitrile/water solution (2:2:1, *v*/*v*/*v*) was added. The samples were then subjected to ultrasonic extraction, incubation, and centrifugation. The supernatants were collected, vacuum-dried, and reconstituted in acetonitrile/water solution prior to injection. Chromatographic separation was performed using a HILIC column, with acetonitrile and an aqueous solution containing 25 mM ammonium acetate and ammonia as mobile phases for gradient elution. Mass spectrometric analysis was performed in both positive and negative electrospray ionization (ESI) modes. When the AB Triple TOF 6600 system (AB SCIEX, Marlborough, MA, USA) was used, primary and secondary mass spectra were acquired in information-dependent acquisition (IDA) mode. When the Orbitrap Exploris™ 480 system (Thermo Fisher Scientific, Waltham, MA, USA) was used, data were acquired in high-resolution mode, with both MS1 and MS2 resolutions set at 60,000. Quality control (QC) samples were inserted throughout the analytical sequence to evaluate system stability. Raw data were converted into mzXML format using ProteoWizard. Peak alignment, retention time correction, and peak extraction were performed using XCMS software 4.10.1. After preprocessing procedures, including missing-value filtering (>50%), K-nearest neighbor (KNN) imputation, and relative standard deviation (RSD) filtering (>50%), metabolite identification and subsequent statistical analyses were conducted. Significantly differential metabolites were screened using the criteria of fold change (FC) > 1.5 or FC < 0.67 and *p* < 0.05.

### 2.7. Targeted Metabolomics Analysis

Targeted metabolomics was further employed to characterize the amino acid composition of porcine ovarian tissues. Amino acid metabolomics analysis was performed by Shanghai Applied Protein Technology Co., Ltd./APTBIO (Shanghai, China). Samples were thawed at 4 °C, and an appropriate amount of each sample was mixed with 400 μL of pre-cooled methanol/acetonitrile solution (1:1, *v*/*v*) containing internal standards. The mixtures were vortexed, subjected to low-temperature ultrasonication for 5 min, and incubated at −20 °C for 1 h to precipitate proteins. Subsequently, the samples were centrifuged at 14,000 rcf for 20 min at 4 °C, and the supernatants were collected and dried under nitrogen gas. Before mass spectrometric analysis, the dried samples were reconstituted in 150 μL of 50% acetonitrile solution, centrifuged again, and the supernatants were collected for injection. Chromatographic separation was performed using an Agilent 1290 Infinity UHPLC system (CA, USA) equipped with a HILIC column. Mass spectrometric detection was conducted using an AB 6500+ QTRAP mass spectrometer (SCIEX, Framingham, MA, USA) operating in positive multiple reaction monitoring (MRM) mode. Calibration curves were established using serially diluted standard solutions. The limits of detection (LOD) and quantification (LOQ) were determined at signal-to-noise ratios of 3:1 and 10:1, respectively. Randomized sample injection and QC sample insertion were used to evaluate system stability, which was assessed by relative standard deviation (RSD). Raw data were processed using MultiQuant 3.0.2 software for peak area extraction and amino acid identification. Significantly differential amino acids were screened according to the criteria of FC > 1.5 or FC < 0.67 and *p* < 0.05.

### 2.8. RNA Extraction and Transcriptomic Analysis

RNA-seq analysis was performed by Shanghai Applied Protein Technology Co., Ltd./APTBIO (Shanghai, China). Immediately after the collection of ovarian tissue samples, TRIzol reagent was added. Total RNA was extracted from the tissues according to the instructions of the TRIzol^®^ reagent protocol (Life Technologies, Carlsbad, CA, USA), and genomic DNA was removed using DNase I (TaKaRa, Kusatsu, Japan). RNA quality was assessed using an Agilent 2100 Bioanalyzer (Agilent Technologies, Santa Clara, CA, USA)., and RNA concentration was quantified using an ND-2000 spectrophotometer (NanoDrop Technologies, Wilmington, DE, USA). High-quality RNA samples, defined as OD260/280 = 1.8–2.0, OD260/230 ≥ 2.0, RNA integrity number (RIN) ≥ 6.5, concentration ≥ 100 ng/μL, and total amount ≥ 2 μg, were used for subsequent library construction and sequencing. Sequencing was performed using the Illumina NovaSeq 6000 platform (Illumina, San Diego, CA, USA). Clean reads were aligned to the reference genome using HISAT2 to obtain their genomic mapping positions. Gene expression levels in each sample were calculated as FPKM values (fragments per kilobase of transcript per million mapped reads) using featureCounts software (version 2.0.3). Differential gene expression analysis was then performed using the DESeq2 software (version 1.40.2, Bioconductor, https://www.bioconductor.org/). Differentially expressed genes were screened according to the criteria of FC > 1.5 or FC < 0.67 and *p* < 0.05.

### 2.9. Protein Extraction and Proteomic Analysis

Proteomic analysis was performed by Shanghai Applied Protein Technology Co., Ltd./APTBIO (Shanghai, China). Proteins were extracted from the samples using the SDT lysis method, with the SDT buffer consisting of 4% (*w*/*v*) SDS, 100 mM Tris/HCl at pH 7.6, and 0.1 M DTT. Protein concentrations were determined using the BCA assay. An appropriate amount of protein from each sample was digested with trypsin using the filter-aided sample preparation (FASP) method. The resulting peptides were desalted using C18 cartridges, lyophilized, and reconstituted in 40 μL of 0.1% formic acid solution. Peptide concentrations were determined by measuring absorbance at OD280. Each sample was separated using an Easy-nLC nanoflow HPLC system. After chromatographic separation, peptides were analyzed using a Q-Exactive mass spectrometer. Database searching, protein identification, and quantitative analysis were performed using MaxQuant software, version 1.5.3.17. Proteins with FC > 1.5 or FC < 0.67 and *p* < 0.05 were defined as significantly differentially expressed proteins.

### 2.10. Phosphoproteomic Analysis

Phosphoproteomic analysis was performed by Shanghai Applied Protein Technology Co., Ltd./APTBIO (Shanghai, China). Proteins were extracted according to the internal standard operating procedures of APTBIO. A total of 15 μg of protein from each sample was subjected to SDS-PAGE to evaluate inter-sample consistency. Proteins were reduced in 8 M urea (UA) buffer with DTT at a final concentration of 20 mM at 37 °C for 1.5 h and alkylated with IAA at a final concentration of 20 mM for 30 min at room temperature in the dark. Subsequently, 100 mM NH_4_HCO_3_ was added to dilute the UA concentration to below 1.5 M. Trypsin digestion was performed at 37 °C for 15–18 h at a trypsin-to-protein ratio of 3:200. After digestion, the reaction was terminated by acidification with 0.1% TFA. Peptides were desalted using C18 solid-phase extraction columns (Empore™ SPE, CDS Analytical, Oxford, PA, USA, 30 μm; Waters) and lyophilized. Phosphorylated peptides were enriched using the High-Select™ Fe-NTA Phosphopeptide Enrichment Kit (Thermo Fisher Scientific, Waltham, MA, USA; catalog no. A32992). The enriched products were vacuum-concentrated and reconstituted in 0.1% formic acid. For each sample, 2 μg of peptides was mixed with iRT standard peptides (Biognosys, Biognosys AG, Schlieren, Switzerland, Ki-3002-2) and subjected to data-independent acquisition (DIA) mass spectrometric analysis. Chromatographic separation was performed using a Vanquish Neo nanoflow high-performance liquid chromatography system, and DIA mass spectrometry was conducted on an Astral high-resolution mass spectrometer (Thermo Scientific). Data processing was performed using Spectronaut software (version 18.5, Biognosys AG, Zurich, Switzerland), and identified proteins were filtered at a false discovery rate (FDR) of <1%. Phosphorylated peptides with FC > 1.5 or FC < 0.67 and *p* < 0.05 were defined as significantly differentially expressed phosphorylated peptides.

### 2.11. Peptidomic Analysis

Peptidomic analysis was performed by Biotree Biomedical Technology Co., Ltd. (Beijing, China). A total of 400 μL of each sample was transferred into a 10 kDa ultrafiltration centrifuge tube and centrifuged at 12,000× *g* for 10 min at 4 °C. The filtrate containing peptides smaller than 10 kDa, approximately 200 μL, was collected, and its concentration was determined using a NanoDrop spectrophotometer. A 100 μL aliquot of the filtrate was reduced with DTT at a final concentration of 10 mM at 56 °C for 1 h, followed by alkylation with IAA at a final concentration of 20 mM for 40 min at room temperature in the dark. Additional DTT was then added to terminate the reaction. The samples were desalted using C18 StageTips and vacuum-dried at 45 °C. LC–MS/MS analysis was performed using a PepMap Neo C18 trap column (300 μm × 5 mm, Thermo Fisher Scientific, Waltham, MA, USA) and an analytical column (150 μm × 170 mm, 1.9 μm, Thermo Fisher Scientific, Waltham, MA, USA). Mobile phase A consisted of 0.1% formic acid, whereas mobile phase B consisted of 0.1% formic acid in 80% acetonitrile. Peptides were separated using a 66 min gradient elution at a flow rate of 600 nL/min. Mass spectrometry was performed in data-dependent acquisition (DDA) mode, with an MS1 resolution of 120,000 and a scan range of 300–1800 *m*/*z*. MS2 spectra were acquired at a resolution of 15,000, with a collision energy of 30 and a cycle time of 2 s. Raw data were searched against the UniProt Sus scrofa protein database. Carbamidomethylation of cysteine was set as a fixed modification, whereas methionine oxidation and protein N-terminal acetylation were set as variable modifications. Enzyme specificity was set as non-specific. The mass tolerances were set to 20 ppm for MS1 and 0.02 Da for MS2. Peptides with a fold change ≥ 2 and *p* < 0.05 were considered significantly different. The screened upregulated peptides were further characterized, and their safety was evaluated using ToxinPred (https://webs.iiitd.edu.in/raghava/toxinpred/, accessed on 17 May 2026) for toxicity prediction and AllerCatPro (https://allercatpro.bii.a-star.edu.sg/, accessed on 17 May 2026) for allergenicity prediction.

### 2.12. Multi-Omics Integration and Candidate Target Selection

Transcriptomic, proteomic, and phosphoproteomic datasets were integrated for multi-omics analysis. Gene and protein identifiers were standardized according to database annotations. Venn diagram analyses were performed among all detected transcripts, differentially expressed transcripts, all identified proteins, and differentially expressed proteins, as well as among all identified proteins, differentially expressed proteins, all identified phosphoproteins, and differentially expressed phosphoproteins. Genes that were detected and differentially expressed at both the mRNA and protein levels were defined as candidate targets, resulting in the identification of nine overlapping targets. Spearman correlation analysis was subsequently performed using their expression profiles across matched samples, and four targets showing significant positive correlations between mRNA and protein abundance (*p* < 0.05) were selected for validation by qRT-PCR and Western blotting. In addition, phosphoproteins were matched to their corresponding parent proteins, and overlapping proteins showing significant changes in either total protein abundance or phosphorylation levels were identified by Venn diagram analysis to assess the consistency and differences between protein expression and phosphorylation modifications.

### 2.13. Quantitative Real-Time PCR

Total RNA was extracted from porcine ovarian tissues using TRIzol reagent. RNA concentration was subsequently determined using a Takara kit (Takara Bio Inc., Kusatsu, Japan). After confirming RNA purity, 2 μg of total RNA was reverse-transcribed using a miRNA first-strand cDNA synthesis kit (Sangon Biotech, Shanghai, China). A 2 μL aliquot of cDNA was diluted 150-fold for quantitative real-time PCR (qPCR). The analysis of differentially expressed miRNAs was performed using specific forward primer sequences designed and synthesized by Sangon Biotech (Shanghai, China). Relative expression levels were normalized to the internal reference gene, and fold changes were calculated using the 2^−ΔΔCt^ method.

### 2.14. Western Blotting

Proteins were extracted from porcine ovarian tissues using a standardized cell lysis buffer. Protein concentrations were determined using an enhanced BCA protein assay kit purchased from Beyotime Biotechnology (Shanghai, China), and the protein samples were diluted to 1 mg/mL for subsequent analysis. Equal amounts of cell lysates, 40 μL per sample, were separated by 10% sodium dodecyl sulfate–polyacrylamide gel electrophoresis (SDS-PAGE) and subsequently transferred onto nitrocellulose membranes (Pall Corporation, Port Washington, NY, USA). The membranes were blocked with 5% skimmed milk powder (BioFroxx, Guangzhou, China) for 1 h at room temperature and then incubated with primary antibodies overnight at 4 °C. After washing, the membranes were incubated with secondary antibodies for 1 h at room temperature. Protein bands were visualized using a chemiluminescence detection system (JS-M6, PeiQing, Shanghai, China) and quantified using ImageJ 1.42q software (National Institutes of Health, Bethesda, MD, USA). All antibodies were diluted in Tris-buffered saline containing Tween 20 (TBST).

### 2.15. Immunofluorescence Staining

Ovarian tissues were fixed in 4% paraformaldehyde, dehydrated through a graded ethanol series, cleared in xylene, embedded in paraffin, and sectioned at a thickness of 4 μm. After deparaffinization in xylene, rehydration through graded ethanol, and washing with PBS, the sections were subjected to antigen retrieval by heating in citrate buffer (pH 6.0). After cooling to room temperature, the sections were washed again with PBS, permeabilized with 0.3% Triton X-100 (Servicebio/Sevier, Wuhan, China) for 10 min at room temperature, and blocked with 5% BSA for 30 min to reduce nonspecific binding. The sections were then incubated with a primary antibody against PPM1K overnight at 4 °C in the dark. On the following day, after washing with PBS, the sections were incubated with a fluorescence-conjugated secondary antibody for 1 h at room temperature in the dark. Nuclei were counterstained with DAPI, and the sections were mounted with antifade mounting medium after PBS washing. Images were captured using a fluorescence microscope. Negative controls were prepared by replacing the primary antibody with PBS, while all other procedures were performed identically. For each sample, five randomly selected fields were photographed under identical imaging parameters. The PPM1K-positive fluorescence signal was semi-quantitatively analyzed using ImageJ software. The integrated optical density (IOD) and positive pixel area were measured, and the mean optical density (MOD) was calculated as IOD/positive pixel area. The average value of five fields from each sample was used as the final value for comparison between the CO and DL groups.

### 2.16. Molecular Docking and Molecular Dynamics Simulation

The three-dimensional structures of MGST2 and PPM1K were obtained from the RCSB Protein Data Bank (PDB), with PDB IDs 6SSS and 4DA1, respectively. After structure retrieval, water molecules and original ligands were removed, and the processed protein structures were saved in PDB format for subsequent molecular docking. Peptide ligand sequences were obtained from peptidomic analysis. The peptides GVEEHETL, GVSEIQQ, and PGIPT were modeled using Avogadro software (version 1.2.0). After preprocessing, molecular docking was performed using the HDOCK server. Protein–peptide complexes were visualized and analyzed using PyMOL (version 2.5.5). To further assess the structural stability of the predicted complexes, molecular dynamics simulations were conducted using GROMACS software (version 2023.2). The amber99sb-ildn force field and TIP3P water model were applied, and the entire system was placed in a water box with dimensions of 7.8 × 7.8 × 7.8 nm. NaCl was added at a concentration of 0.15 M, and additional Na^+^ or Cl^−^ ions were added as needed to neutralize the system. Energy minimization was performed using the steepest descent algorithm. The system was equilibrated for 400 ps under each of the NVT and NPT ensembles, with the temperature maintained at 300 K and the pressure during NPT equilibration set to 0.1 MPa. All bonds were constrained using the LINCS algorithm, and long-range electrostatic interactions were calculated using the particle mesh Ewald (PME) method. The van der Waals cutoff radius was set to 1.4 nm. The simulation time step was 2 fs, and trajectory data were saved every 10 ps. A 100 ns molecular dynamics simulation was performed for each free protein and the corresponding protein–peptide complex under these conditions.

### 2.17. Statistical Analysis

All data are presented as the mean ± standard error (SE). Statistical analyses were performed using SPSS version 26.0. Differences between the two groups in the sow experiment were analyzed using Student’s *t*-test. For the mouse biosafety experiment, differences among groups were analyzed using one-way analysis of variance (ANOVA), followed by the least significant difference (LSD) test for pairwise comparisons. Bar graphs were generated using GraphPad Prism version 8.0.2. A *p* < 0.05 was considered statistically significant. For targeted metabolomics, untargeted metabolomics, transcriptomics, proteomics, phosphoproteomics, and peptidomics analyses, *p*-values were adjusted for multiple comparisons using the Benjamini–Hochberg false discovery rate (FDR) method. Features with an adjusted *p*-value < 0.05 were considered significantly different.

## 3. Results

### 3.1. Nutrient and Amino Acid Composition of Periplaneta americana and PAR

The nutrient and amino acid compositions of dried whole *Periplaneta americana* and PAR are presented in Table 2 and Table 3. Both materials had high crude protein contents, reaching 68.31% in the dried whole insect and 65.04% in PAR. Following ethanol extraction, the crude fat content decreased from 33.03% to 16.88%, whereas the crude fiber content increased from 6.12% to 7.84%. PAR contained 7.03% moisture, 2.69% ash, 0.21% calcium, and 0.31% phosphorus. PAR also exhibited a diverse amino acid profile, with glutamic acid being the most abundant amino acid at 5.34%, followed by aspartic acid (3.92%), alanine (3.70%), proline (3.19%), glycine (3.03%), and tyrosine (3.00%). Compared with the dried whole insect, PAR contained higher levels of most of the detected amino acids, particularly histidine and proline, which increased from 1.14% and 2.26% to 2.55% and 3.19%, respectively. Compared with imported fish meal, the crude protein content is similar; PAR contained lower levels of most amino acids, but higher levels of tyrosine, histidine, and proline. Overall, these results indicate that PAR retains a high protein content and a diverse amino acid composition after ethanol extraction, supporting its potential as an alternative protein ingredient in animal feed.

### 3.2. Biosafety Evaluation of Dietary PAR in Mice

**Figure 1:**
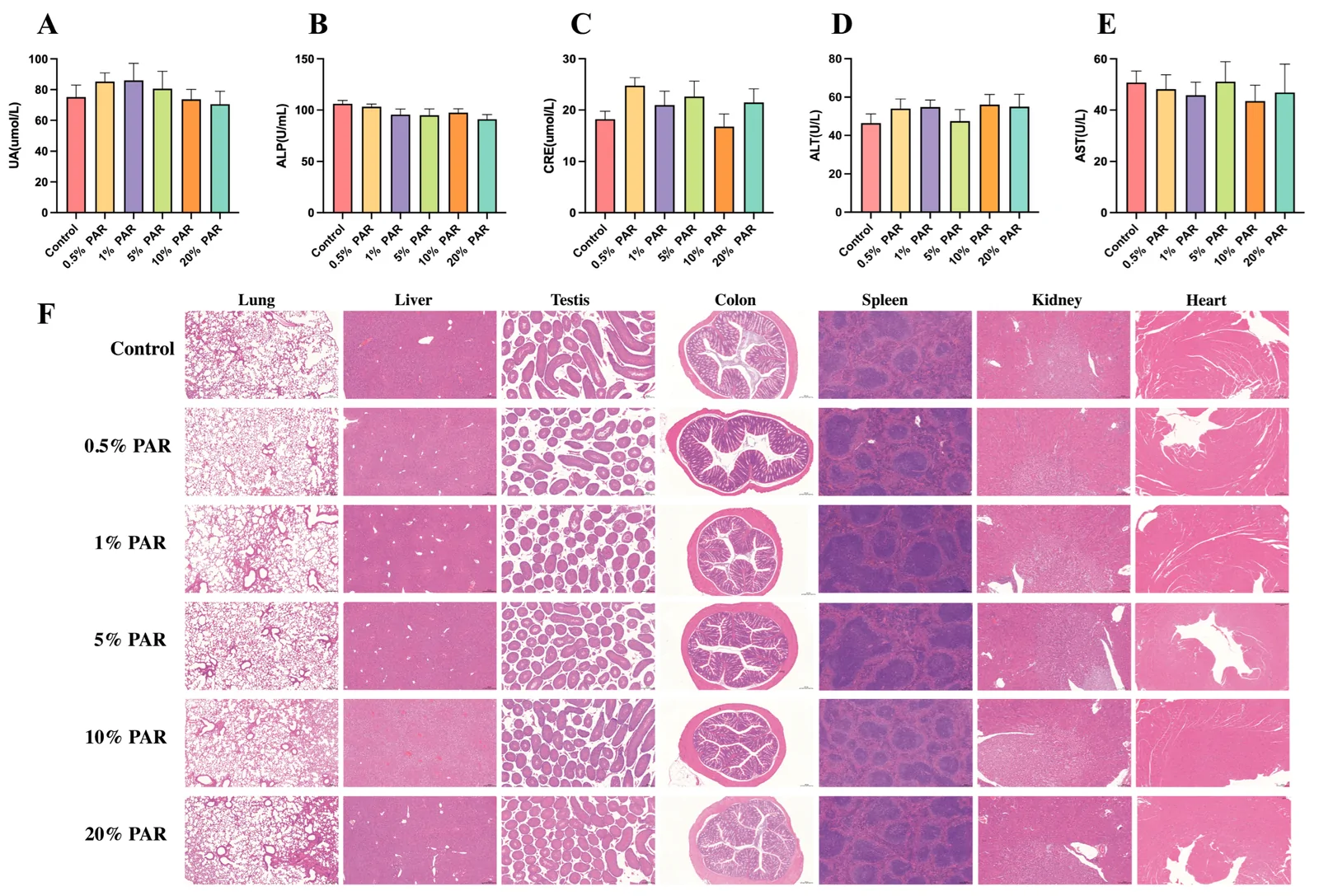
Effects of different dietary inclusion levels of defatted *Periplaneta americana* on biochemical parameters and the histomorphology of major organs in mice. (**A**) Uric acid (UA) level; (**B**) alkaline phosphatase (ALP) activity; (**C**) creatinine (CRE) level; (**D**) aspartate aminotransferase (AST) activity; (**E**) alanine aminotransferase (ALT) activity; and (**F**) hematoxylin and eosin (H&E) staining of the heart, liver, spleen, lung, kidney, testis, and colon of mice in the different treatment groups. *n* = 6; no significant differences were observed between any treatment group and the control group (*p* > 0.05).

As shown in Figure 1A–E, dietary supplementation with 0.5%, 1%, 5%, 10%, or 20% PAR for 28 days had no significant effects on serum UA and CRE levels or ALP, AST, and ALT activities compared with the control diet (*p* > 0.05). Histopathological examination further showed that the heart, liver, spleen, lung, kidney, testis, and colon maintained normal tissue architecture in all PAR-supplemented groups, with no apparent lesions, structural abnormalities, or cellular damage observed relative to the control group (Figure 1F). These results indicate that dietary PAR supplementation at inclusion levels of up to 20% did not cause detectable biochemical or histopathological toxicity in mice during the 28-day feeding period.

### 3.3. Effects of PAR on Sow Ovaries

**Figure 2:**
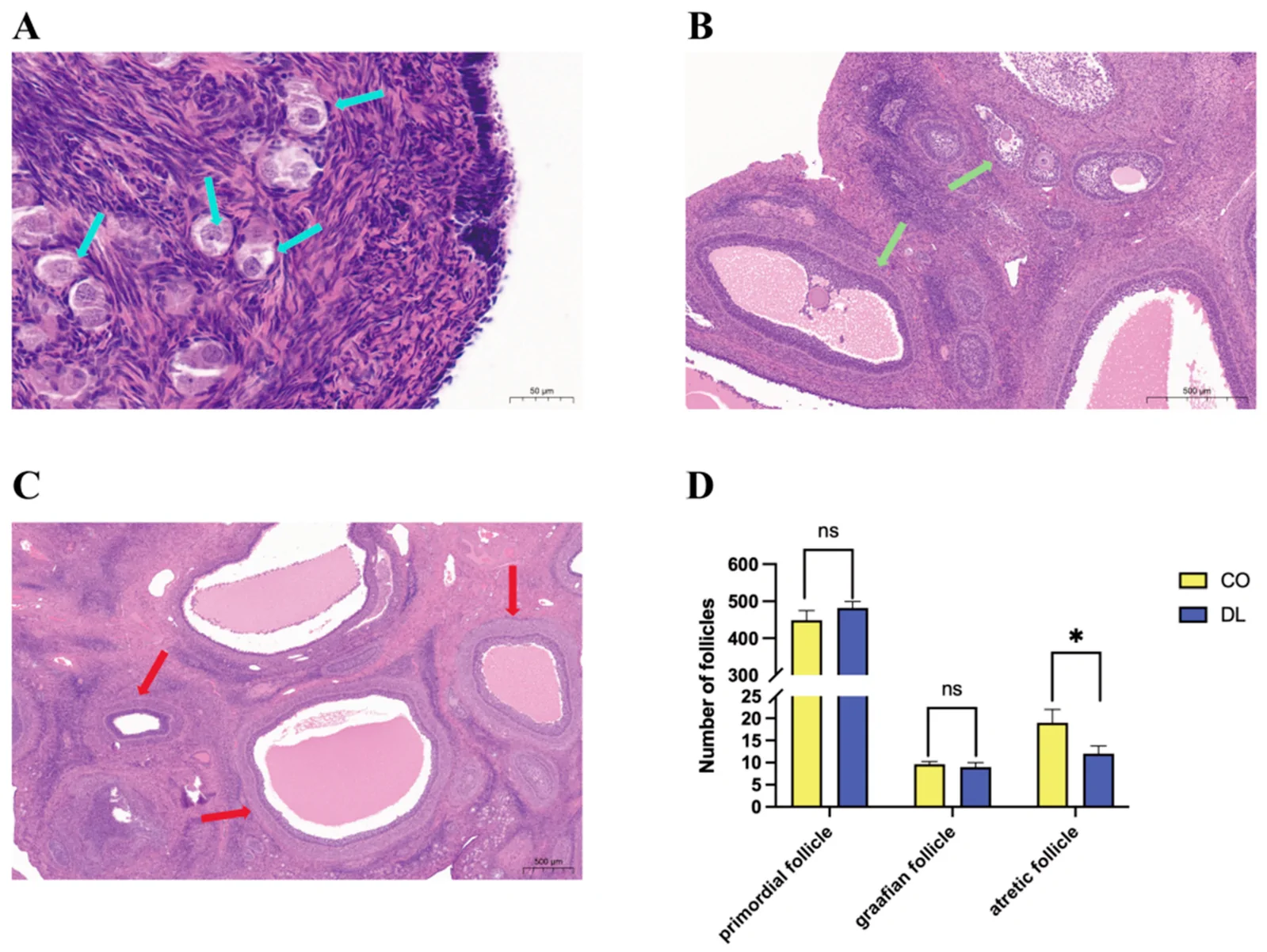
Histological morphology of ovarian tissue and statistical analysis of follicle numbers. (**A**) primordial follicle; (**B**) Graafian follicle; (**C**) atretic follicle; (**D**) quantitative analysis of different follicle types in the CO and DL groups. ns indicates no significant difference; *n* = 3, * indicates *p* < 0.05.

H&E staining was performed to evaluate ovarian histological morphology and follicular development in the CO and DL groups. As shown in Figure 2A–C, primordial follicles, Graafian follicles, and atretic follicles were clearly observed in ovarian tissue sections. Quantitative analysis showed that the number of primordial follicles was slightly higher in the DL group than in the CO group, whereas no significant difference was observed between the two groups (*p* > 0.05). Similarly, the number of Graafian follicles showed no significant difference between the CO and DL groups (*p* > 0.05). Notably, the number of atretic follicles was significantly decreased in the DL group compared with the CO group (*p* < 0.05) (Figure 2D). These results suggest that dietary supplementation with PAR may reduce follicular atresia in sow ovaries without markedly affecting the numbers of primordial or Graafian follicles.

### 3.4. Effects of Dietary PAR Supplementation on Growth Performance, Ovarian Development, and Serum Biochemical Indices

**Figure 3:**
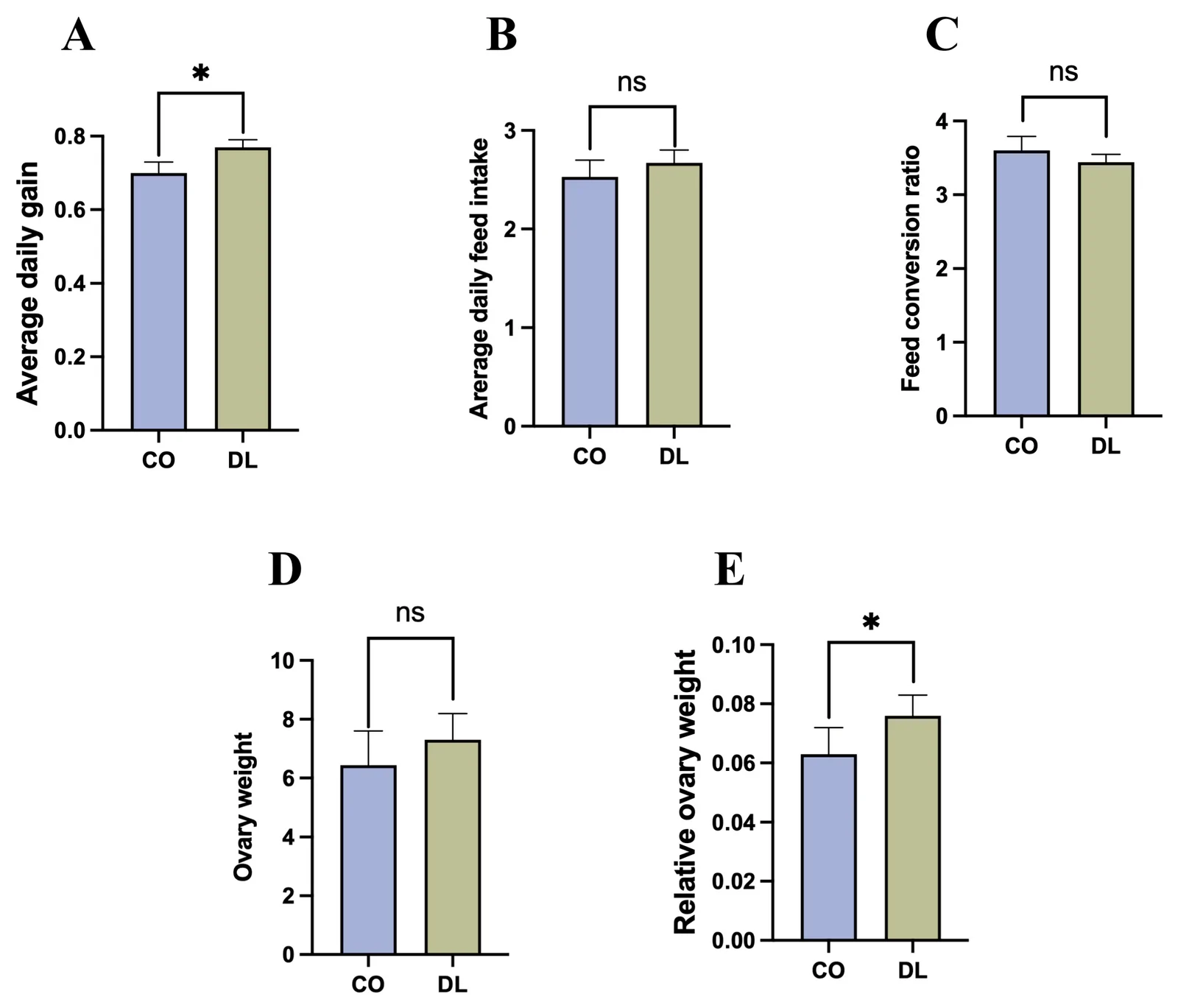
Effects of dietary defatted *Periplaneta americana* supplementation on growth performance and ovarian indices in pigs. (**A**) Average daily gain (ADG); (**B**) average daily feed intake (ADFI); (**C**) feed conversion ratio (FCR); (**D**) ovary weight; and (**E**) relative ovary weight. CO, control group; DL, diet supplemented with 3% defatted *P. americana*. For growth performance parameters (**A**–**C**), *n* = 15 per group; for ovarian indices (**D**,**E**), *n* = 5, * indicates *p* < 0.05; ns, not significant.

As shown in Figure 3A–C, dietary supplementation with 3% PAR significantly increased the average daily gain of pigs. In contrast, no significant differences were observed in average daily feed intake or feed conversion ratio between the CO and DL groups, indicating that PAR supplementation promoted growth without significantly affecting feed intake or feed conversion efficiency. Regarding ovarian development, ovarian weight was numerically higher in the DL group than in the CO group, although the difference was not statistically significant. However, the relative ovary weight was significantly increased in the DL group, suggesting that dietary PAR supplementation promoted relative ovarian development (Figure 3D,E). Serum biochemical analysis showed that the MDA level was significantly lower in the DL group than in the CO group, indicating reduced lipid peroxidation (Table 4). No significant differences were observed between the two groups in T-AOC, T-SOD, GSH-Px, or CAT activities or in the levels of the inflammatory cytokines TNF-α, IL-1β, and IL-6. Collectively, these findings indicate that dietary supplementation with 3% PAR improved growth performance, increased relative ovary weight, and alleviated lipid peroxidation without adversely affecting feed utilization, systemic antioxidant enzyme activities, or inflammatory status.

### 3.5. Identification and Comparative Analysis of Differential Metabolites

**Figure 4:**
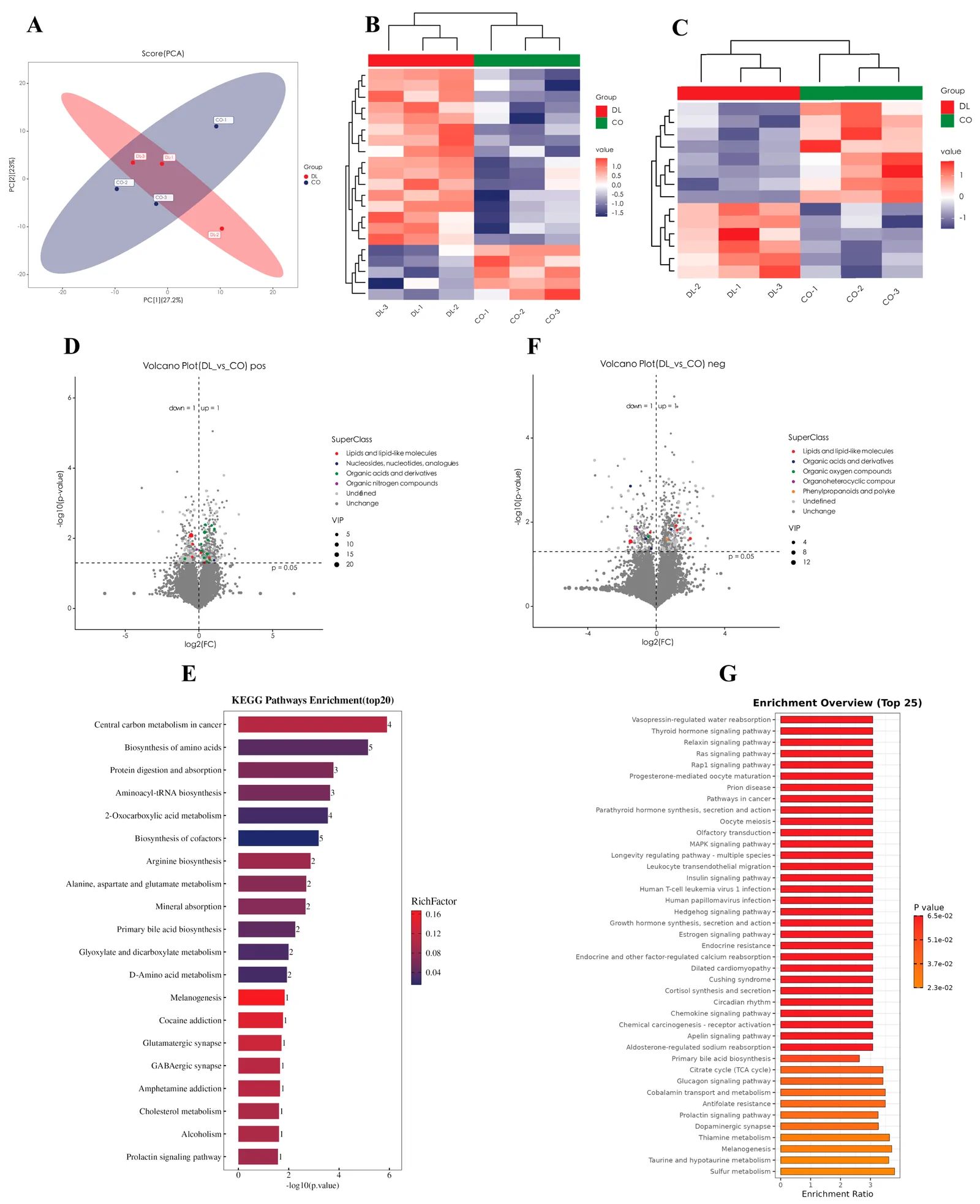
Differentially accumulated metabolites (DAMs) between the CO and DL groups. (**A**) Principal component analysis (PCA). (**B**) Heatmap of differentially accumulated metabolites (DAMs) in positive ion mode. (**C**) Heatmap of DAMs in negative ion mode. (**D**) Volcano plot of DAMs in positive ion mode. (**E**) Volcano plot of DAMs in negative ion mode. (**F**) KEGG pathway enrichment analysis. (**G**) MSEA pathway enrichment analysis.

To investigate the effects of dietary PAR supplementation on the metabolic characteristics of porcine ovarian tissues, untargeted metabolomic analysis was performed on ovarian samples from the CO and DL groups. Principal component analysis (PCA) showed a certain degree of separation between the two groups in the principal component space, indicating that dietary PAR supplementation induced alterations in the overall metabolic profile of ovarian tissues (Figure 4A). Further hierarchical clustering analysis of differential metabolites revealed distinct clustering patterns between the CO and DL groups under both positive and negative ion modes. Samples within the same group clustered relatively well, suggesting marked differences in the abundance patterns of differential metabolites between the two groups (Figure 4B,C). In positive ion mode, a total of 21 differential metabolites were identified, including 16 upregulated and 5 downregulated metabolites. In the negative ion mode, 14 differential metabolites were identified, including 6 upregulated and 8 downregulated metabolites. As shown in the volcano plots (Figure 4D,E), these differential metabolites mainly included lipids and lipid-like molecules, organic acids and derivatives, organic oxygen compounds, nucleosides, nucleotides, and their analogues, suggesting that dietary PAR supplementation may affect lipid metabolism, nucleotide metabolism, and energy metabolism in ovarian tissues. To further elucidate the biological functions associated with these differential metabolites, the metabolites identified under positive and negative ion modes were combined for KEGG enrichment analysis. As shown in Figure 4F, the enriched pathways were mainly involved in energy metabolism, amino acid synthesis and transport, redox homeostasis, lipid and cholesterol metabolism, and endocrine regulation, indicating that dietary PAR supplementation may participate in the regulation of ovarian function by modulating substance metabolism and hormone-related pathways in ovarian tissues. In addition, metabolite set enrichment analysis (MSEA) showed that the differential metabolites were associated with multiple pathways related to ovarian development, hormonal signaling, and cellular functional regulation (Figure 4G). Among these pathways, progesterone-mediated oocyte maturation, oocyte meiosis, estrogen signaling, and GnRH signaling are closely associated with follicular development, oocyte maturation, and ovarian endocrine function, whereas mTOR, Ras, and insulin signaling are involved in cell proliferation, energy sensing, and nutritional metabolism regulation. Notably, the enrichment of pathways such as the citrate cycle (TCA cycle), taurine and hypotaurine metabolism, sulfur metabolism, and glutathione metabolism further suggests that dietary PAR supplementation may influence mitochondrial energy metabolism, sulfur-containing amino acid metabolism, and antioxidant defense processes in ovarian tissues. Together with the decreased serum MDA level observed in the DL group, these findings suggest that PAR may provide a favorable metabolic microenvironment for maintaining ovarian cellular function and supporting follicular development by improving metabolic homeostasis and redox balance in ovarian tissues.

### 3.6. Identification and Comparative Analysis of Amino Acids in Porcine Ovarian Samples

**Figure 5:**
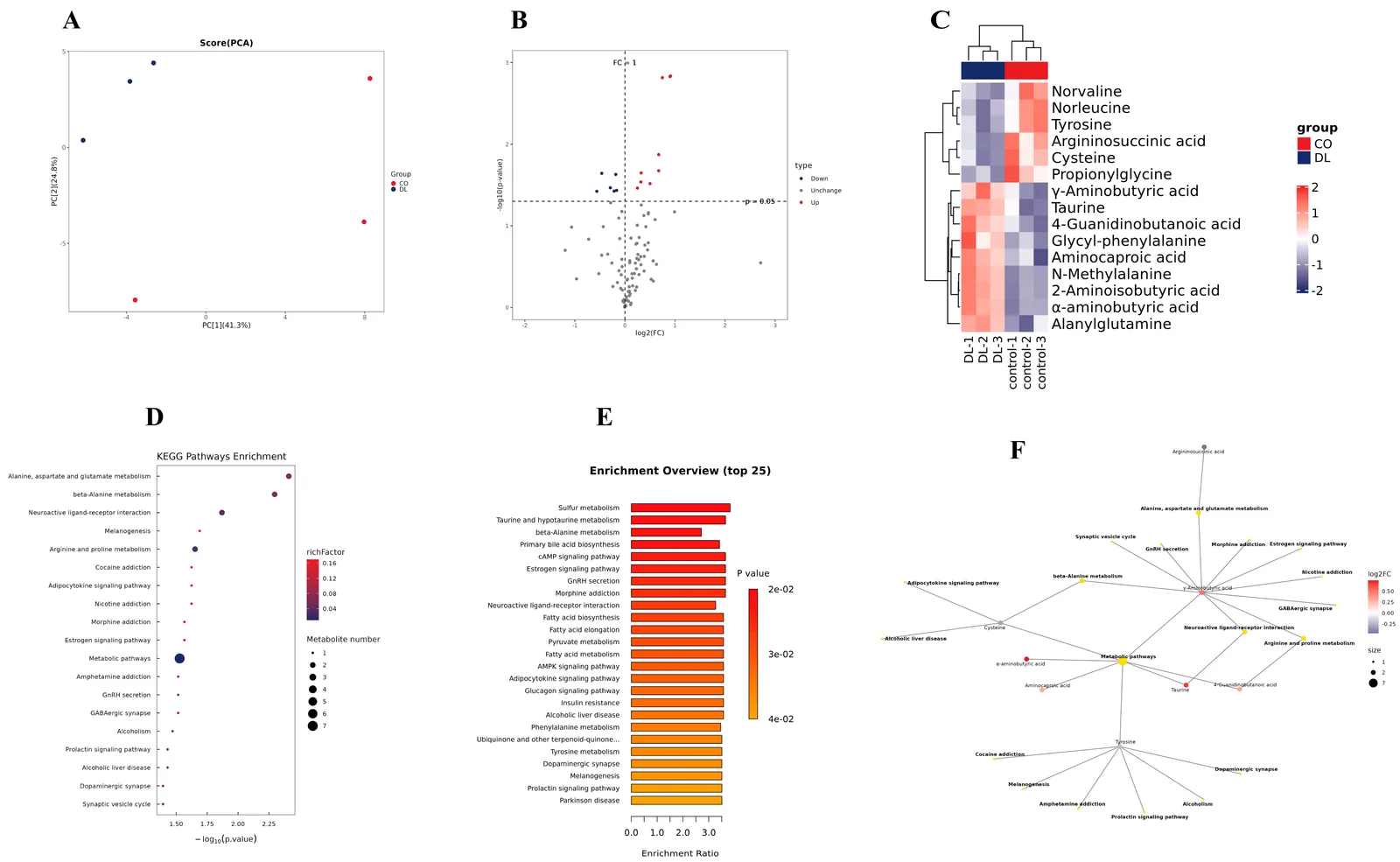
Differential amino acid metabolites between the CO and DL groups. (**A**) Principal component analysis (PCA). (**B**) Volcano plot of differential amino acid metabolites. (**C**) Heatmap of differential amino acid metabolites. (**D**) KEGG pathway enrichment analysis. (**E**) MSEA pathway enrichment analysis. (**F**) Metabolite–pathway association network.

The untargeted metabolomic results revealed significant enrichment of amino acid metabolism-related pathways. Therefore, to further explore the effects of dietary PAR supplementation on amino acid metabolism in sow ovarian tissues, an amino acid-targeted metabolomic analysis was performed on ovarian samples from the CO and DL groups to identify differentially accumulated metabolites (DAMs). A total of 99 metabolites were identified. PCA showed a clear separation between the CO and DL groups in the principal component space, indicating pronounced differences in the amino acid metabolic profiles of ovarian tissues between the two groups (Figure 5A,B). Based on clustering analysis of DAMs, 15 differential metabolites were identified between the CO and DL groups, including 9 upregulated and 6 downregulated metabolites (Figure 5C). In the DL group, the levels of 2-aminoisobutyric acid, N-methylalanine, α-aminobutyric acid, alanylglutamine, taurine, γ-aminobutyric acid, 4-guanidinobutanoic acid, aminocaproic acid, and glycyl-phenylalanine were significantly increased. As shown in Figure 5D, KEGG enrichment analysis indicated that the differential amino acid-related metabolites were mainly enriched in alanine, aspartate, and glutamate metabolism, beta-alanine metabolism, biosynthesis of amino acids, arginine and proline metabolism, cysteine and methionine metabolism, estrogen signaling pathway, cortisol synthesis and secretion, and glutathione metabolism. These pathways are primarily involved in amino acid metabolism, neuroendocrine regulation, estrogen signaling, transmembrane transport, and redox homeostasis, suggesting that dietary PAR supplementation may affect ovarian tissue function by regulating amino acid metabolic networks and hormone-related signaling pathways. To compensate for the limitations of KEGG enrichment analysis in metabolite enrichment and to further identify low-abundance metabolites with potential biological regulatory significance and their associated metabolic pathways, MSEA was further performed. Alterations in pathways such as sulfur metabolism and taurine and hypotaurine metabolism were also identified, as shown in Figure 5E. Among the enriched pathways, the estrogen signaling pathway, GnRH secretion, oxytocin signaling pathway, prolactin signaling pathway, and AMPK signaling pathway suggest that dietary PAR supplementation may influence ovarian development and function in sows by modulating hormonal changes and energy metabolism. A network analysis was performed to elucidate the relationships between differential metabolites and metabolic pathways, as shown in Figure 5F. Multiple differential amino acid-related metabolites were found to form a complex association network with several enriched pathways. In this network, γ-aminobutyric acid, tyrosine, taurine, cysteine, and argininosuccinic acid were connected with multiple metabolic or signaling pathways, suggesting that these metabolites may serve as important nodes through which dietary PAR regulates the ovarian amino acid metabolic network. In particular, taurine was closely associated with taurine and hypotaurine metabolism, sulfur metabolism, and antioxidant-related processes; γ-aminobutyric acid was associated with neuroactive ligand–receptor interactions and endocrine regulation; and tyrosine may participate in hormone synthesis and cellular signal transduction.

### 3.7. Identification and Comparative Analysis of Differentially Expressed Genes

**Figure 6:**
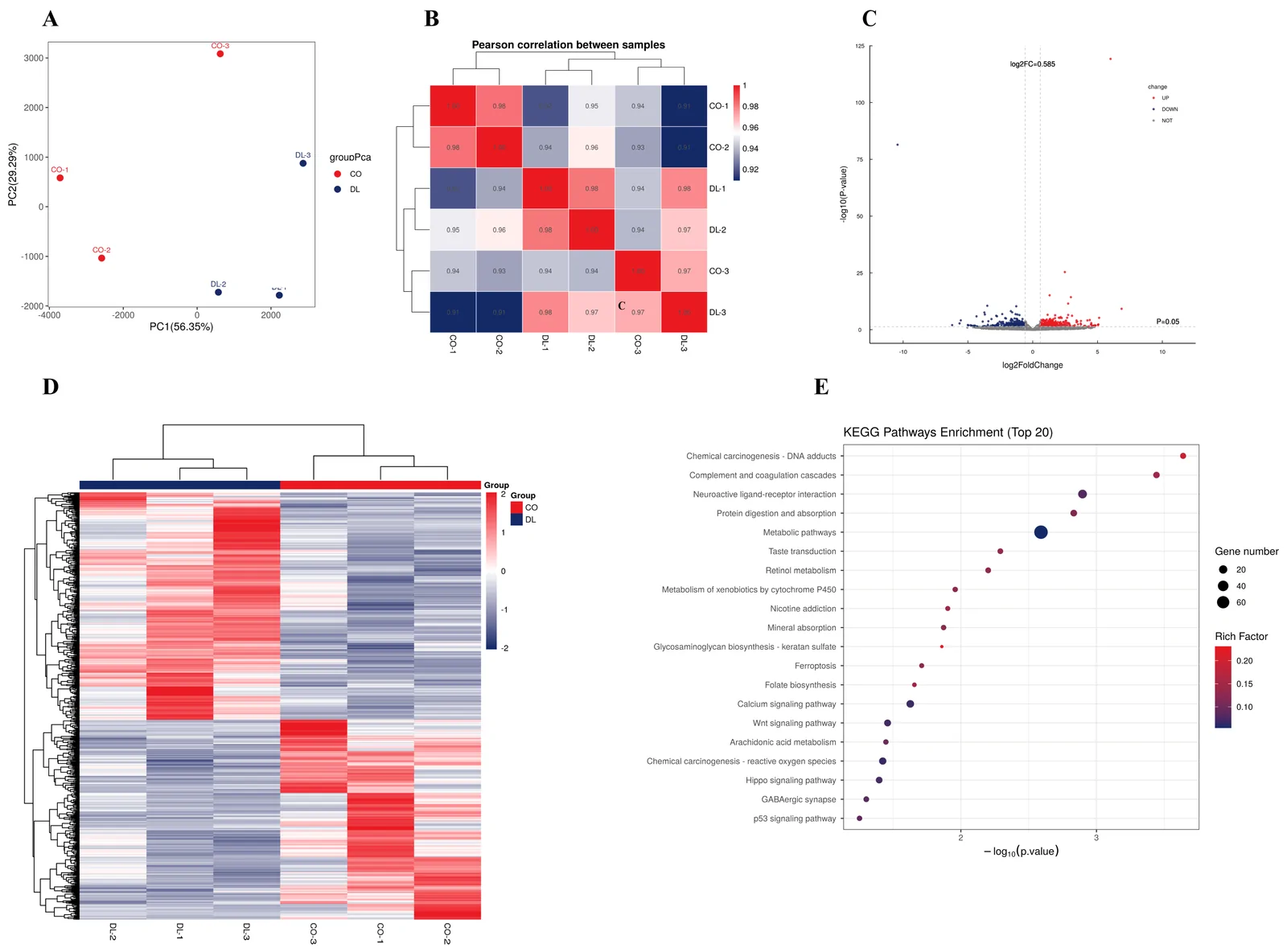
Transcriptomic analysis of differentially expressed genes (DEGs) in ovarian samples from the CO and DL groups. (**A**) Principal component analysis (PCA). (**B**) Pearson correlation coefficient (PCC) plot of identified mRNAs. (**C**) Volcano plot of DEGs. (**D**) Heatmap of DEGs. (**E**) KEGG pathway enrichment analysis.

To investigate the effects of dietary PAR supplementation on the gene expression profile of porcine ovarian tissues, transcriptome sequencing was performed on ovarian samples from the CO and DL groups. Principal component analysis (PCA) revealed a clear separation between the two groups in the principal component space, indicating that dietary PAR supplementation altered the global transcriptional expression pattern of porcine ovarian tissues (Figure 6A). Pearson correlation analysis showed high correlations among samples within the same group, suggesting good biological reproducibility and demonstrating the stability and reliability of the transcriptomic sequencing data (Figure 6B). A total of 24,356 genes were detected by RNA-seq. Differential expression analysis identified 874 differentially expressed genes (DEGs) between the DL and CO groups, including 465 upregulated and 409 downregulated genes (Figure 6C). Hierarchical clustering analysis based on the DEGs further showed distinct expression patterns between the DL and CO groups (Figure 6D). To functionally annotate these 874 DEGs, KEGG enrichment analyses were performed. KEGG enrichment analysis showed that the DEGs were mainly enriched in pathways including chemical carcinogenesis-DNA adducts, complement and coagulation cascades, neuroactive ligand–receptor interaction, protein digestion and absorption, metabolic pathways, retinol metabolism, ferroptosis, calcium signaling pathway, Wnt signaling pathway, arachidonic acid metabolism, Hippo signaling pathway, and p53 signaling pathway (Figure 6E). These pathways are primarily involved in nutrient absorption and metabolism, oxidative stress and detoxification metabolism, ferroptosis, cell fate regulation, cell proliferation and differentiation, neuroendocrine signaling, and ovarian development-related signal transduction. Among them, ferroptosis, Wnt signaling, Hippo signaling, p53 signaling, and calcium signaling are closely associated with oxidative stress, apoptosis, cell proliferation, follicular development, and ovarian tissue homeostasis, suggesting that dietary PAR supplementation may influence ovarian function by regulating cell fate and redox homeostasis. Collectively, these results suggest that dietary PAR supplementation may participate in the regulation of porcine ovarian function by modulating transmembrane transport, ion homeostasis, nutrient metabolism, oxidative stress, the extracellular microenvironment, and signaling pathways such as Wnt, Hippo, p53, and ferroptosis.

### 3.8. Identification and Comparative Analysis of Differentially Abundant Proteins

**Figure 7:**
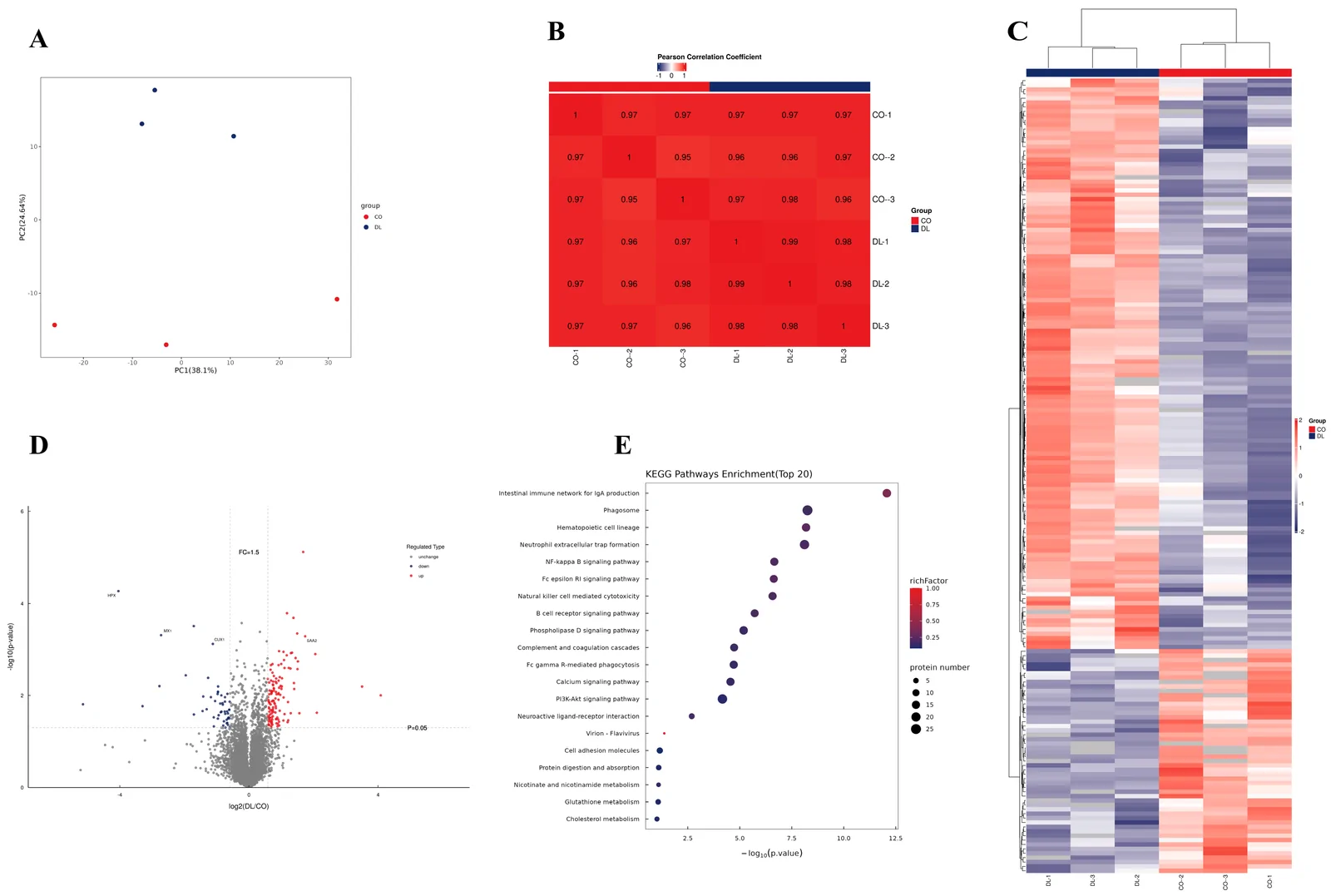
Proteomic analysis of differentially abundant proteins (DAPs) in ovarian samples from the CO and DL groups. (**A**) Principal component analysis (PCA). (**B**) Pearson correlation coefficient (PCC) plot of identified proteins. (**C**) Heatmap of DAPs. (**D**) Volcano plot of DAPs; (**E**) KEGG pathway enrichment analysis.

Proteomic analysis was performed to identify differentially abundant proteins (DAPs) in sow ovarian tissues. PCA revealed a clear separation between the two groups, indicating marked differences in protein expression profiles (Figure 7A). Pearson correlation analysis showed good biological reproducibility among samples within the same group (Figure 7B). Hierarchical clustering analysis based on the DAPs further demonstrated obvious differences between the experimental and control groups (Figure 7C). A total of 7482 proteins were identified, among which 181 DAPs were detected between the DL and CO groups, including 130 upregulated and 51 downregulated proteins (Figure 7D). To further elucidate the reproductive and metabolic processes associated with these DAPs, KEGG enrichment analyses were conducted. The combined KEGG enrichment results showed that the DAPs were mainly involved in immune regulation, inflammatory responses, phagocytosis, oxidative stress, detoxification metabolism, signal transduction, and nutrient metabolism-related processes (Figure 7E). KEGG enrichment analysis indicated that the DAPs were significantly enriched in the intestinal immune network for IgA production, phagosome, neutrophil extracellular trap formation, NF-kappa B signaling pathway, complement and coagulation cascades, Fc gamma R-mediated phagocytosis, calcium signaling pathway, PI3K-Akt signaling pathway, and glutathione metabolism. These enrichment results suggest that dietary PAR supplementation may affect the functional status of porcine ovarian tissues mainly by regulating the ovarian immune microenvironment, inflammatory homeostasis, phagocytic clearance, antioxidant defense, and cellular signal transduction.

### 3.9. Phosphoproteomic Analysis

**Figure 8:**
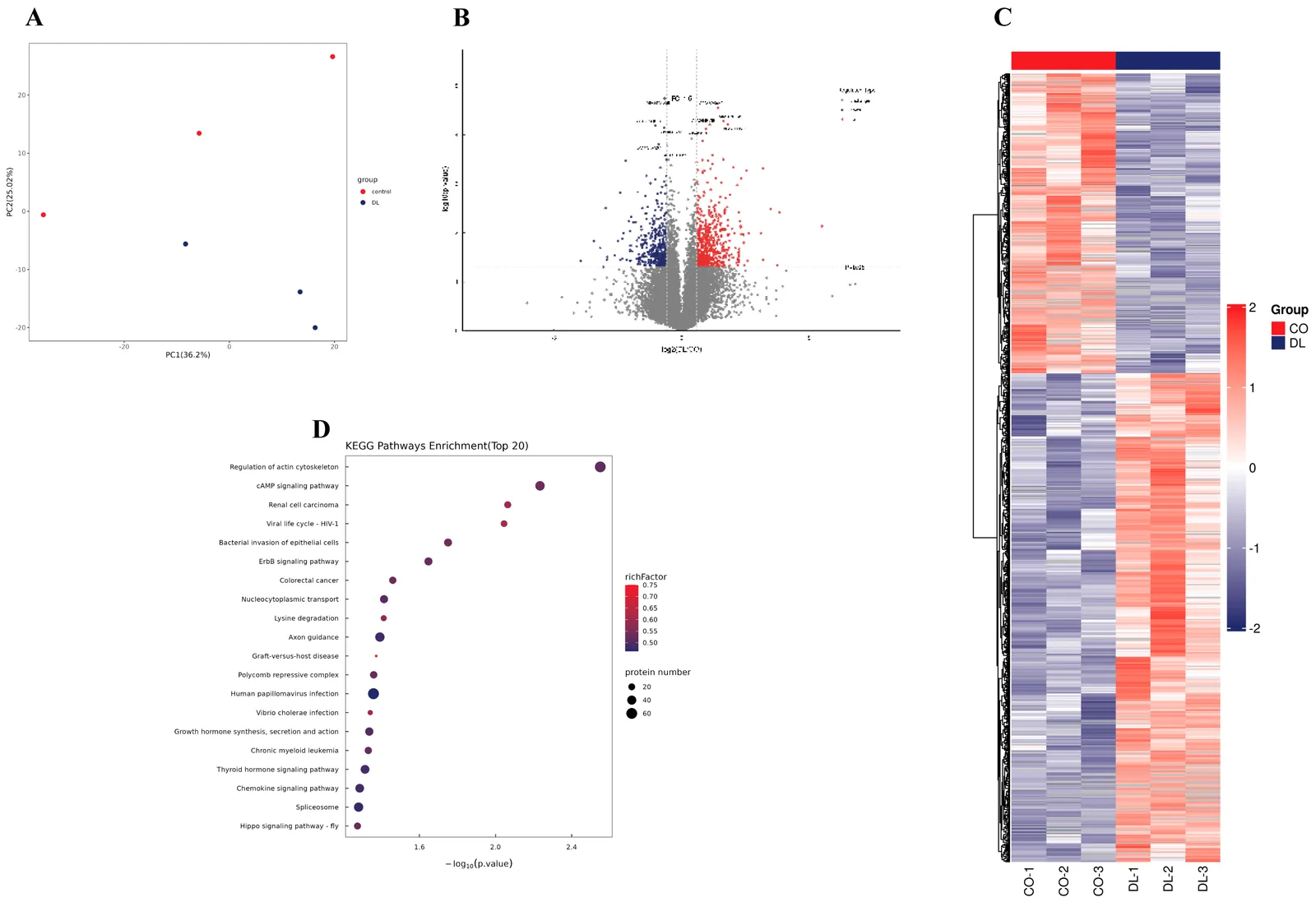
Phosphoproteomic analysis of differentially expressed phosphorylated peptides in ovarian samples from the CO and DL groups. (**A**) Principal component analysis (PCA). (**B**) Volcano plot of differentially expressed phosphorylated peptides. (**C**) Heatmap of differentially expressed phosphorylated peptides. (**D**) KEGG pathway enrichment analysis.

Phosphoproteomic analysis was performed to identify phosphorylated proteins in sow ovarian tissues. PCA showed an apparent separation between the two groups, with PC1 and PC2 explaining 38.2% and 24.64% of the total variance, respectively, indicating significant differences in phosphorylated protein expression profiles (Figure 8A). As shown in Figure 8B, a total of 5400 phosphorylated proteins were identified. Because individual proteins may contain multiple phosphorylation modification sites, and different sites may exhibit distinct upregulation or downregulation trends, unified quantification at the modified protein level was not feasible. Therefore, quantification was performed at the modified peptide level. Hierarchical clustering analysis based on phosphorylated peptides revealed clear differences between the experimental and control groups (Figure 8C). A total of 23,460 phosphorylated peptides were identified, among which 960 differentially phosphorylated peptides were detected between the experimental and control groups, including 595 upregulated and 365 downregulated peptides. KEGG enrichment analyses were then performed on the differentially phosphorylated peptides. KEGG enrichment analysis showed that the differentially phosphorylated peptides were enriched in pathways including regulation of actin cytoskeleton, cAMP signaling pathway, ErbB signaling pathway, growth hormone synthesis, secretion and action, thyroid hormone signaling pathway, chemokine signaling pathway, and Hippo signaling pathway (Figure 8D). These results suggest that dietary PAR supplementation may affect cytoskeletal dynamics, hormone-mediated signal transduction, cellular stress responses, cell proliferation and differentiation, and follicular development-related processes in ovarian tissues by regulating protein phosphorylation modifications.

**Figure 9:**
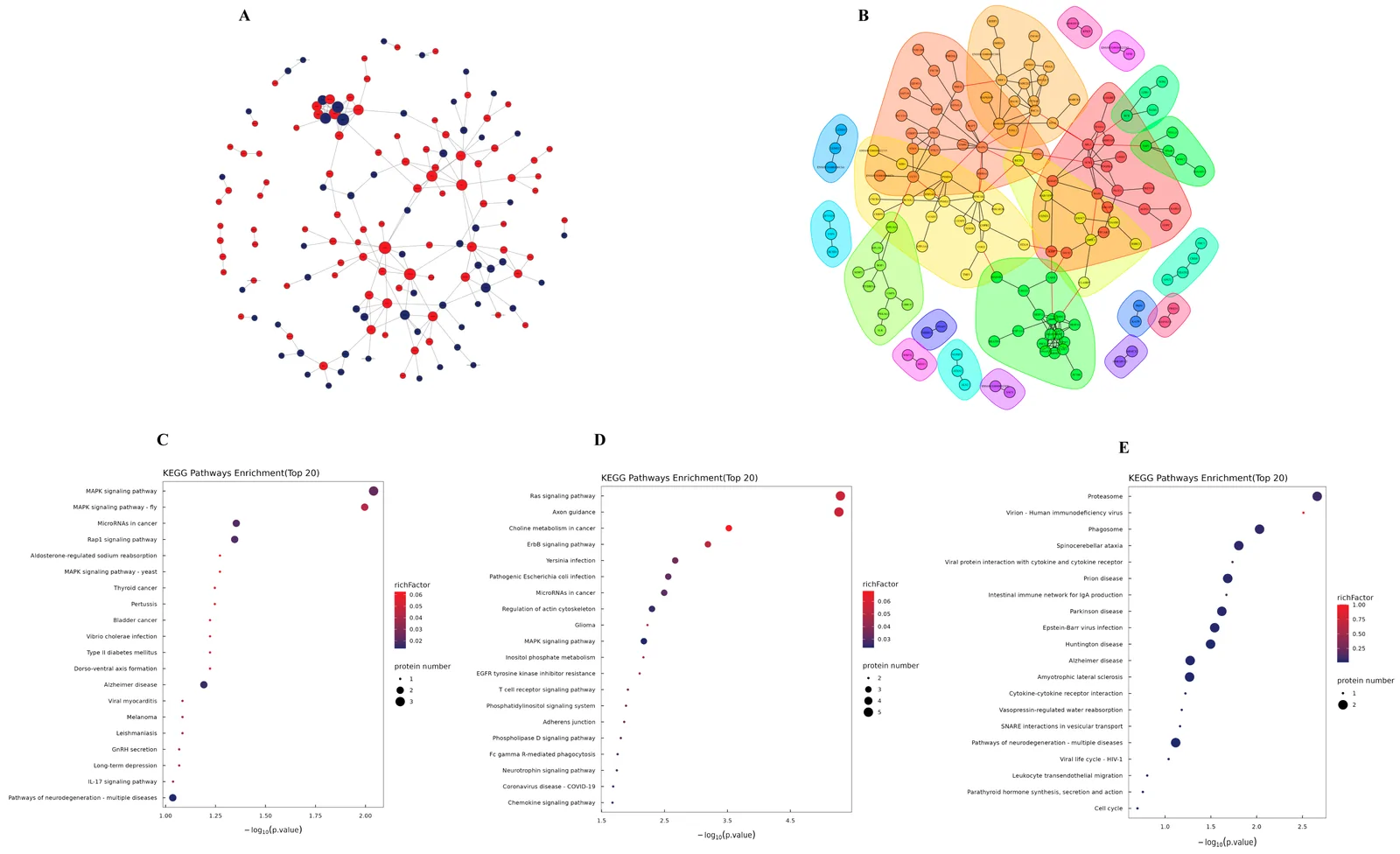
Protein–protein interaction (PPI) network analysis of differentially phosphorylated proteins in ovarian samples from the CO and DL groups. (**A**) PPI network of differentially phosphorylated proteins. (**B**) Functional cluster classification of the PPI network. (**C**–**E**) KEGG pathway enrichment analysis of highly connected proteins in the top three functional clusters.

To investigate the interactions among phosphorylated proteins, a protein–protein interaction (PPI) network was constructed using Cytoscape software (version 3.10.3) based on protein interaction relationships from the STRING database for proteins corresponding to the differentially modified peptides in the comparison group (Figure 9A). In the PPI network, highly clustered proteins generally possess identical or similar biological functions and may exert biological effects through coordinated interactions. Therefore, based on topological clustering principles, modified proteins with high degrees of aggregation in the interaction network were divided into different clusters, followed by functional classification of each cluster. The specific clusters are shown in Figure 9B. According to the functional classification results of highly clustered proteins shown in Figure 9B, multiple clusters were ranked by size, and the top three functional clusters were selected. KEGG enrichment analysis was performed for the highly clustered proteins within each cluster, and KEGG enrichment bubble plots were generated (Figure 9C–E). KEGG enrichment analysis of the major clusters showed that the associated proteins were mainly enriched in the MAPK signaling pathway, Rap1 signaling pathway, AMPK signaling pathway, Ras signaling pathway, ErbB signaling pathway, regulation of actin cytoskeleton, insulin signaling pathway, adherens junction, phospholipase D signaling pathway, chemokine signaling pathway, phagosome, cytokine-cytokine receptor interaction, leukocyte transendothelial migration, and cell cycle. These pathways are mainly involved in cell proliferation and differentiation, energy metabolism, cytoskeletal remodeling, cell adhesion, immune responses, inflammatory homeostasis, and cell cycle regulation. Together with the overall phosphoproteomic enrichment results, these findings indicate that dietary PAR supplementation may participate in the functional regulation of porcine ovarian tissues by modulating MAPK, Ras, ErbB, AMPK, cytoskeleton-related, and immune-related phosphorylation signaling networks.

### 3.10. Serum Peptidomic Analysis

**Figure 10:**
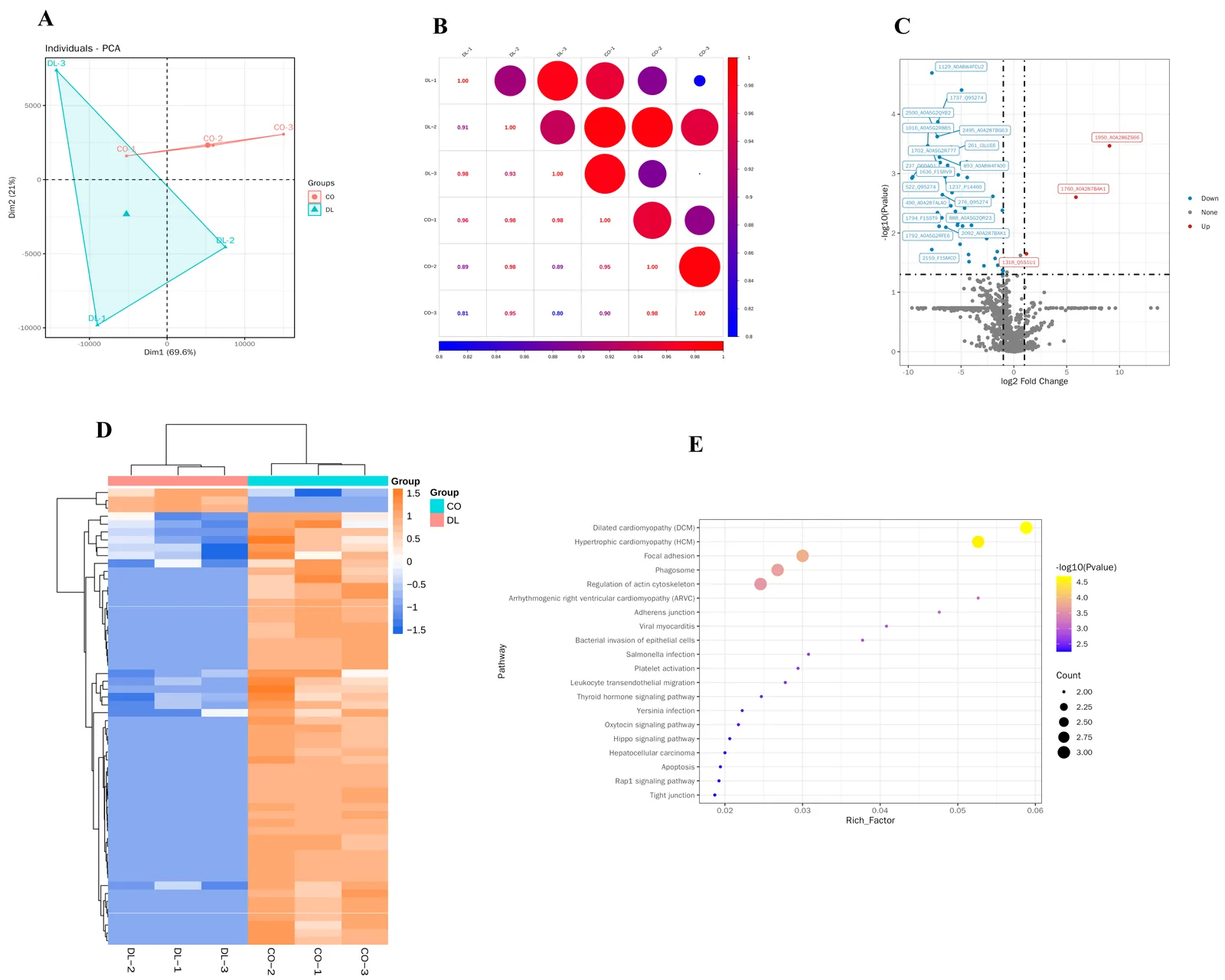
Serum peptidomic analysis of differentially expressed peptides between the CO and DL groups. (**A**) Principal component analysis (PCA). (**B**) Pearson correlation coefficient (PCC) plot of identified peptides. (**C**) Volcano plot of differentially expressed peptides. (**D**) Heatmap of differentially expressed peptides. (**E**) KEGG pathway enrichment analysis.

Serum peptidomic analysis showed that pigs fed the PAR-supplemented diet exhibited a markedly distinct peptide expression profile compared with the control group. PCA and correlation analyses indicated clear separation between the two groups and good intra-group reproducibility (Figure 10A,B). A total of 1465 peptides were identified, among which 58 significantly differential peptides were detected between the DL and CO groups, including 3 upregulated and 55 downregulated peptides (Figure 10C). Hierarchical clustering analysis further confirmed that dietary treatment generated a stable and characteristic peptide expression pattern (Figure 10D). KEGG enrichment analysis showed that the differential serum peptide-derived proteins were significantly enriched in focal adhesion, regulation of actin cytoskeleton, phagosome, adherens junction, platelet activation, leukocyte transendothelial migration, thyroid hormone signaling pathway, oxytocin signaling pathway, Hippo signaling pathway, and apoptosis (Figure 10E). The combined KEGG enrichment results suggest that dietary PAR supplementation may exert systemic regulatory effects on porcine ovarian function through circulating peptide-related endocrine regulation, cytoskeletal remodeling, cell junction homeostasis, immune defense, and apoptosis regulation. In addition, computational tools were used to predict the toxicity and allergenicity of GVSEIQQ, GVEEHETL, and PGIPT. All three peptides were classified as non-toxic and non-allergenic by the prediction tools (Table 5). Antioxidant potential was assessed using AnOxPePred, with GVEEHETL yielding the highest predicted free radical-scavenging score among the three peptides (Table 5). These computational predictions require experimental validation and do not establish peptide safety or antioxidant activity.

### 3.11. Screening and Comparative Analysis of Key Targets

**Figure 11:**
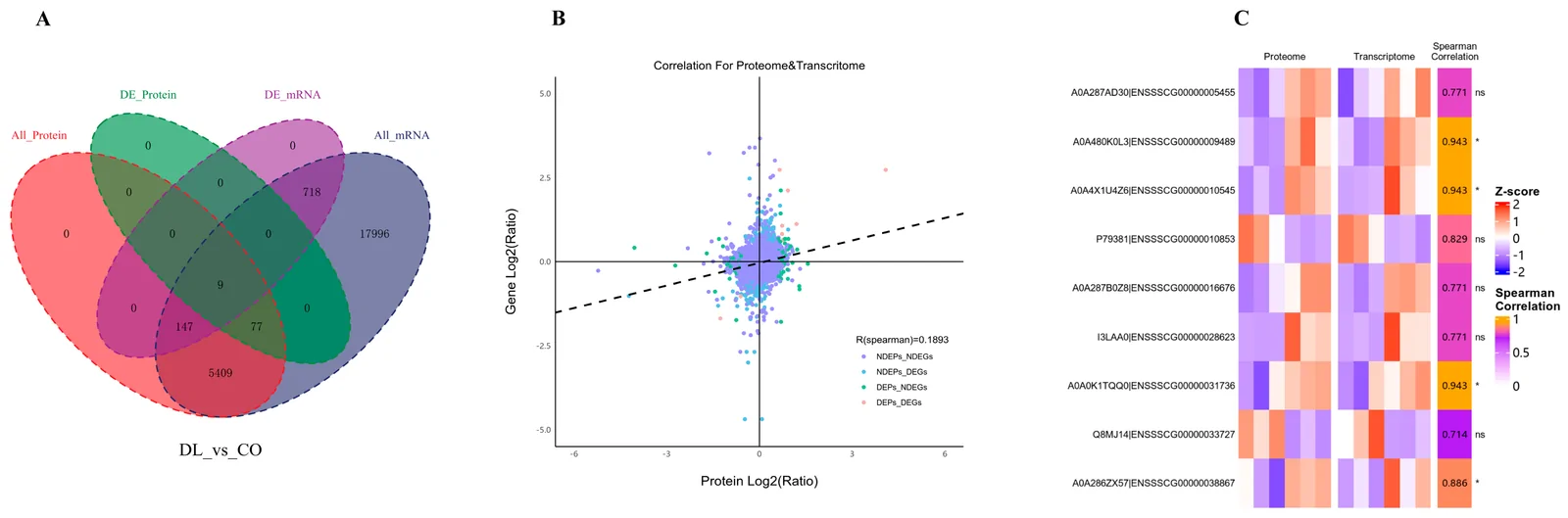
Integrative analysis of transcriptomic and proteomic data in ovarian samples from the CO and DL groups. (**A**) Venn diagram showing the overlap between quantified transcripts/proteins and differentially expressed transcripts/proteins. (**B**) Correlation scatter plot of expression levels between quantified proteins and their corresponding genes. (**C**) Expression association plot of differentially expressed genes and differentially abundant proteins. * indicates *p* < 0.05; ns indicates no statistically significant correlation (*p* ≥ 0.05).

Through integrated transcriptomic and proteomic analysis, nine key targets were identified, namely RARRES1, GPX1, INMT, EPHX1, GPC6, PPM1K, SVEP1, CPN1, and MGST2 (Figure 11A,B). Spearman correlation analysis was used to evaluate the relationship between mRNA expression levels and protein abundance. The results showed that GPC6, CPN1, MGST2, and PPM1K exhibited relatively high correlations between the transcriptomic and proteomic datasets, as shown in Figure 11C.

### 3.12. Validation of Key Targets by Quantitative Real-Time PCR and Western Blotting

**Figure 12:**
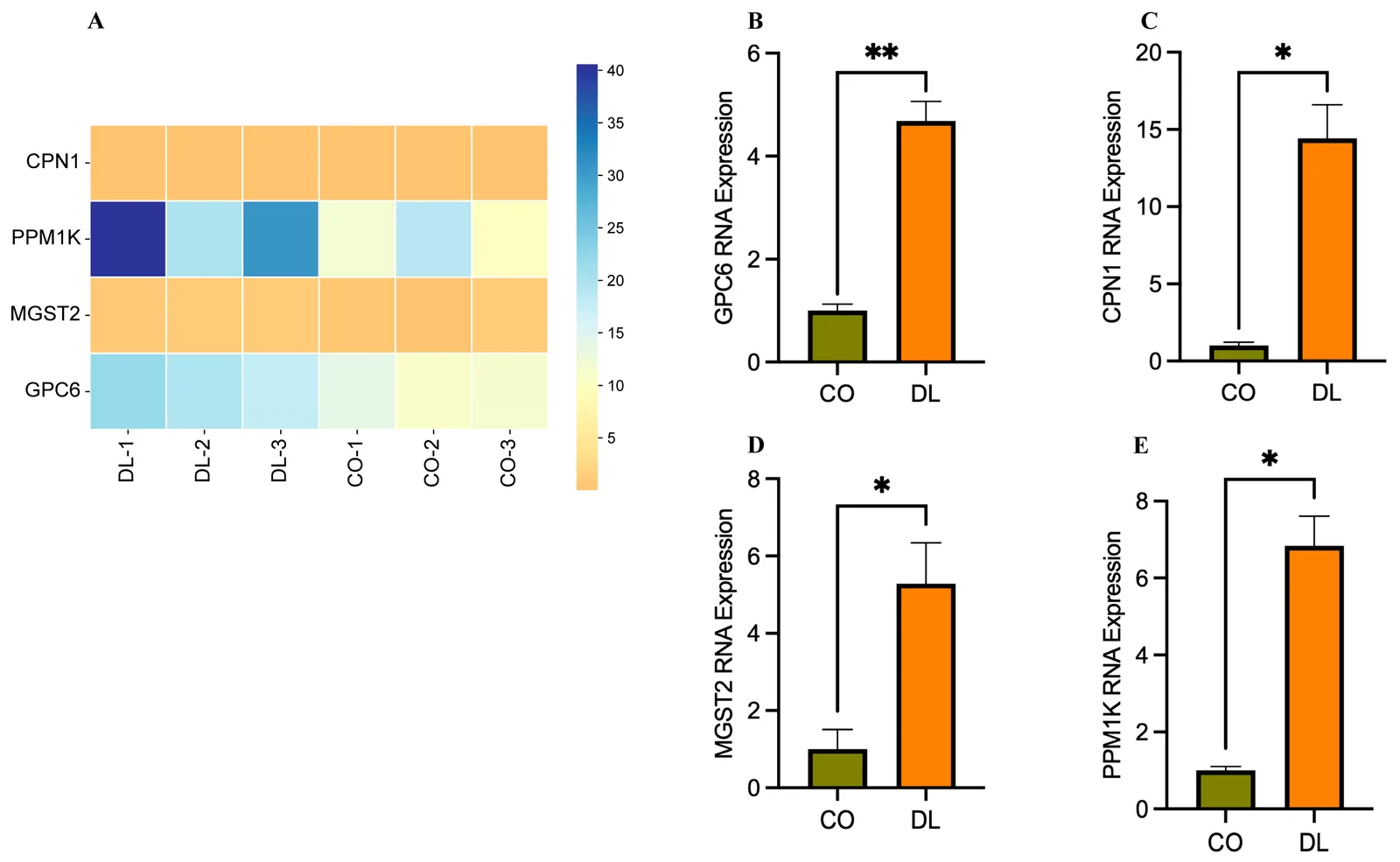
(**A**) Heatmap of candidate gene expression in the CO and DL groups; relative mRNA expression of (**B**) GPC6, (**C**) CPN1, (**D**) MGST2, and (**E**) PPM1K (*n* = 3). * indicates *p* < 0.05; ** indicates *p* < 0.01.

**Figure 13:**
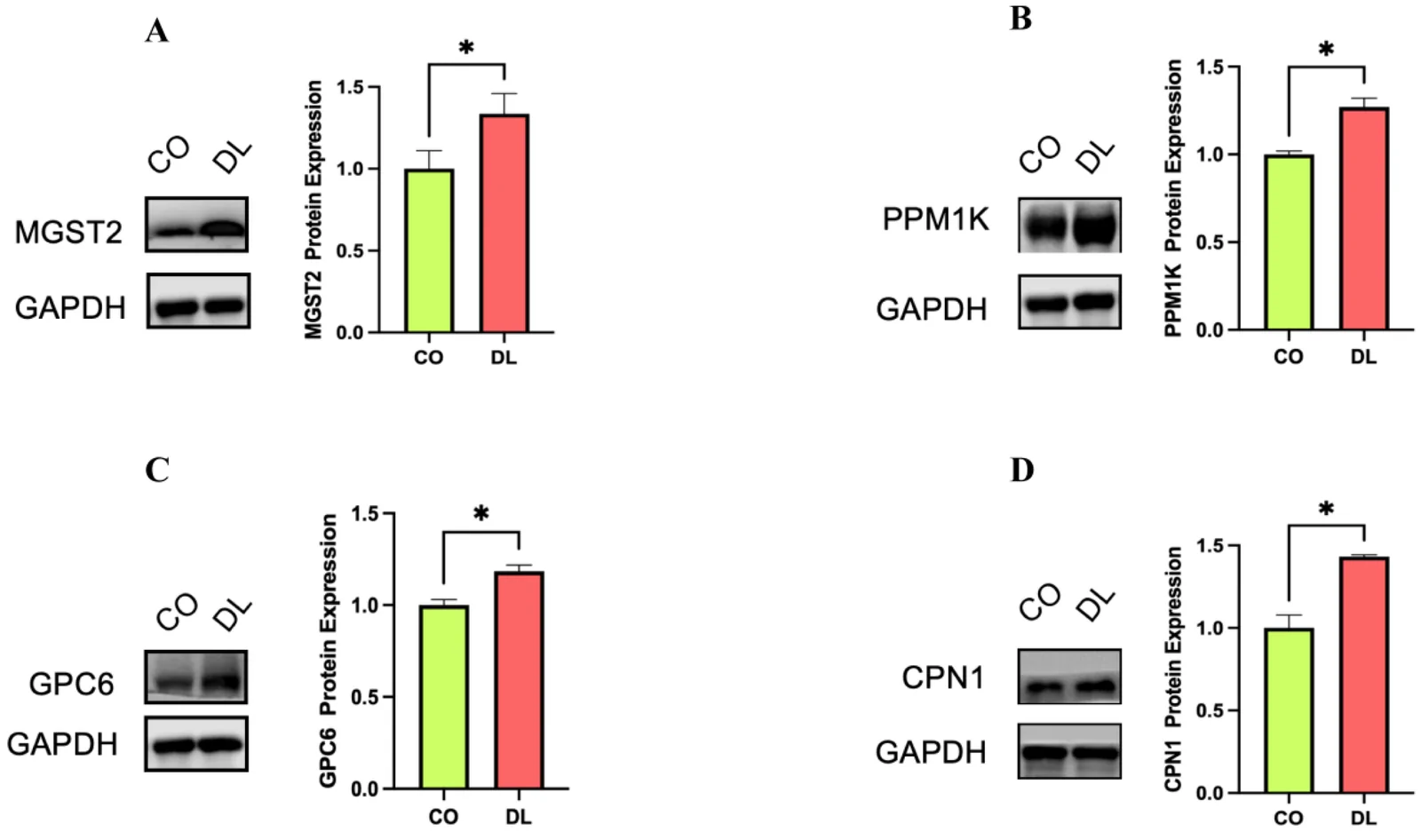
Western blot bands and quantitative analysis of (**A**) MGST2, (**B**) PPM1K, (**C**) GPC6, and (**D**) CPN1 in ovarian tissues from the CO and DL groups. (*n* = 3). * indicates *p* < 0.05.

Among the nine key targets, four genes and their corresponding proteins showing strong concordance between the transcriptomic and proteomic data were selected for validation by qPCR and Western blotting. The expression profiles of these four genes across the sequencing samples are shown in Figure 12A, and the corresponding primer sequences are listed in Table 6. Compared with the control group, GPC6, CPN1, MGST2, and PPM1K were all upregulated in the experimental group. The qPCR results were consistent with the RNA-seq findings (Figure 12B–E). Similarly, the Western blotting results were consistent with the proteomic analysis (Figure 13A–D).

### 3.13. Integrated Analysis of Proteomics and Phosphoproteomics

**Figure 14:**
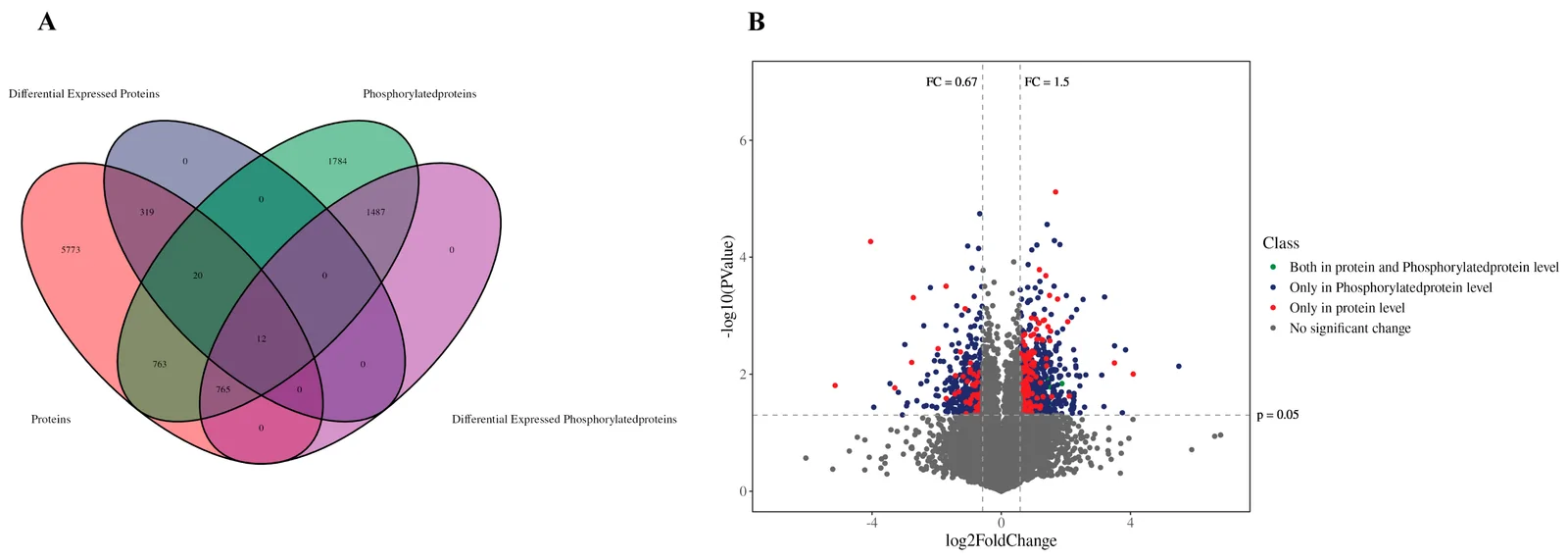
Integrative analysis of proteomic and phosphoproteomic data in ovarian samples from the CO and DL groups. (**A**) Venn diagram of overlapping proteins identified in the proteomic and phosphoproteomic datasets. (**B**) Volcano plot of expression differences in the overlapping proteins.

Integrated analysis of the proteome and modified proteome was performed to systematically characterize changes at the protein level and to identify potential key protein molecules or modification sites associated with biological phenotypic alterations. A Venn diagram identified 12 overlapping proteins showing differential changes at both the protein expression level and the protein modification level (Figure 14A). Further screening using strict thresholds in volcano plot analysis revealed that four of these overlapping proteins, namely HRG, PPM1K, ITIH1, and GPRIN1, exhibited significant differences at both the protein expression level and the modified peptide level (Figure 14B).

### 3.14. Immunofluorescence Validation of PPM1K in Ovarian Tissues

**Figure 15:**
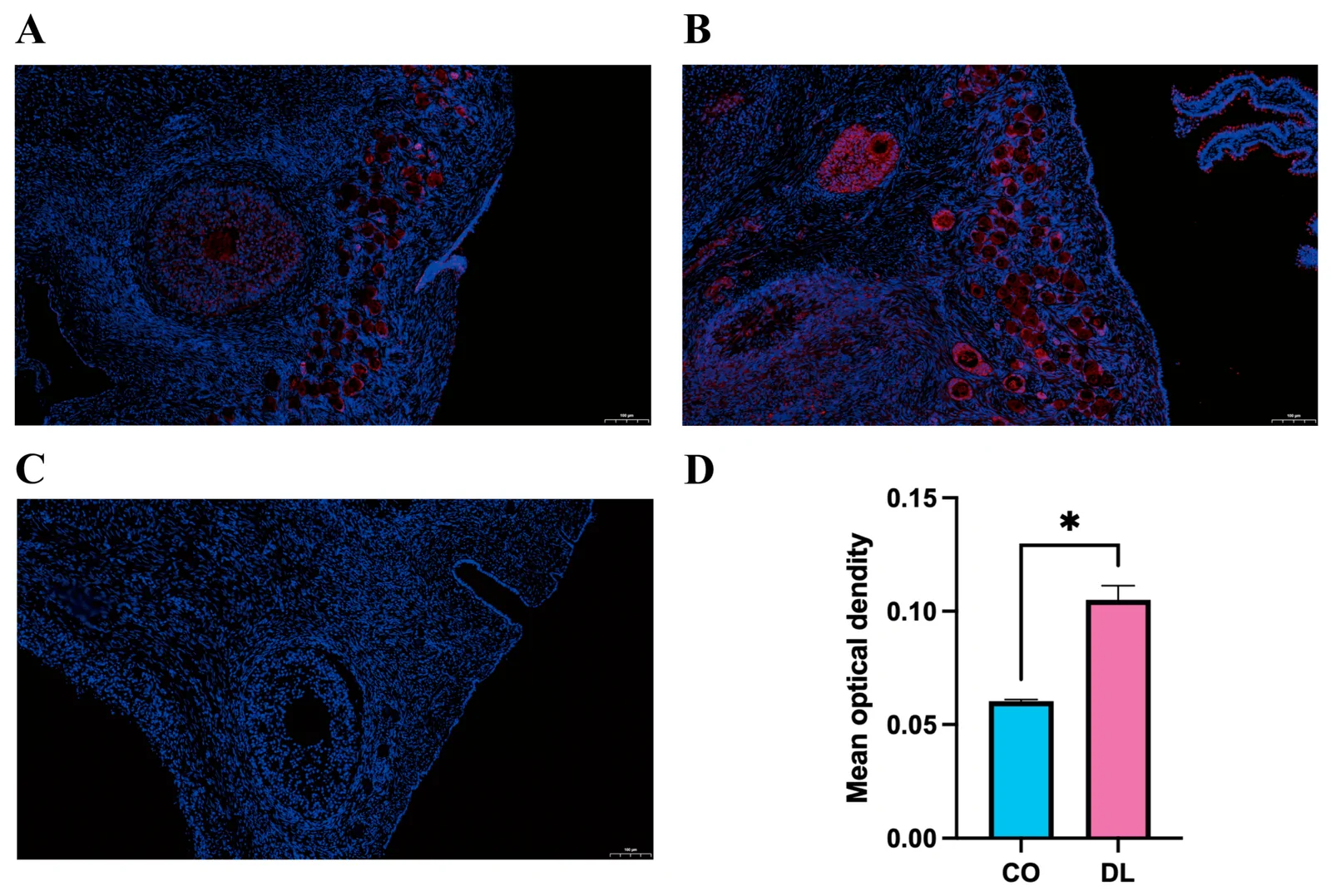
Immunofluorescence staining and quantitative analysis of PPM1K in sow ovarian tissues. (**A**) Immunofluorescence staining of PPM1K in ovarian tissues from the CO group. (**B**) Immunofluorescence staining of PPM1K in ovarian tissues from the DL group. (**C**) Negative control for immunofluorescence staining in ovarian tissues. (**D**) Quantitative analysis of the mean optical density of PPM1K-positive fluorescence in the CO and DL groups (*n* = 3). * indicates *p* < 0.05.

PPM1K showed strong relevance in the integrated transcriptomic, proteomic, and phosphoproteomic analyses. To further validate the expression changes in PPM1K in ovarian tissues, immunofluorescence staining was performed to examine its expression and distribution in ovarian tissues from the CO and DL groups. The results showed that PPM1K-positive red fluorescence signals were observed in ovarian tissues from both groups, and the signals were mainly distributed in ovarian tissue cells. Compared with the CO group, the DL group exhibited markedly enhanced PPM1K-positive fluorescence signals (Figure 15A,B). No obvious specific red fluorescence signal was observed in the negative control, indicating low nonspecific background and good staining specificity (Figure 15C). Furthermore, semi-quantitative analysis of PPM1K-positive fluorescence signals based on mean optical density showed that the mean optical density of PPM1K was significantly higher in the DL group than in the CO group (*p* < 0.05) (Figure 15D). These results indicate that dietary supplementation with PAR upregulates PPM1K expression in sow ovarian tissues. Together with the transcriptomic, proteomic, qPCR, and Western blot validation results, the expression pattern of PPM1K showed good consistency, suggesting that PPM1K may be involved in PAR-mediated regulation of ovarian amino acid metabolism, mitochondrial function, and energy metabolic homeostasis.

### 3.15. Molecular Docking and Molecular Dynamics Simulation Analysis

**Figure 16:**
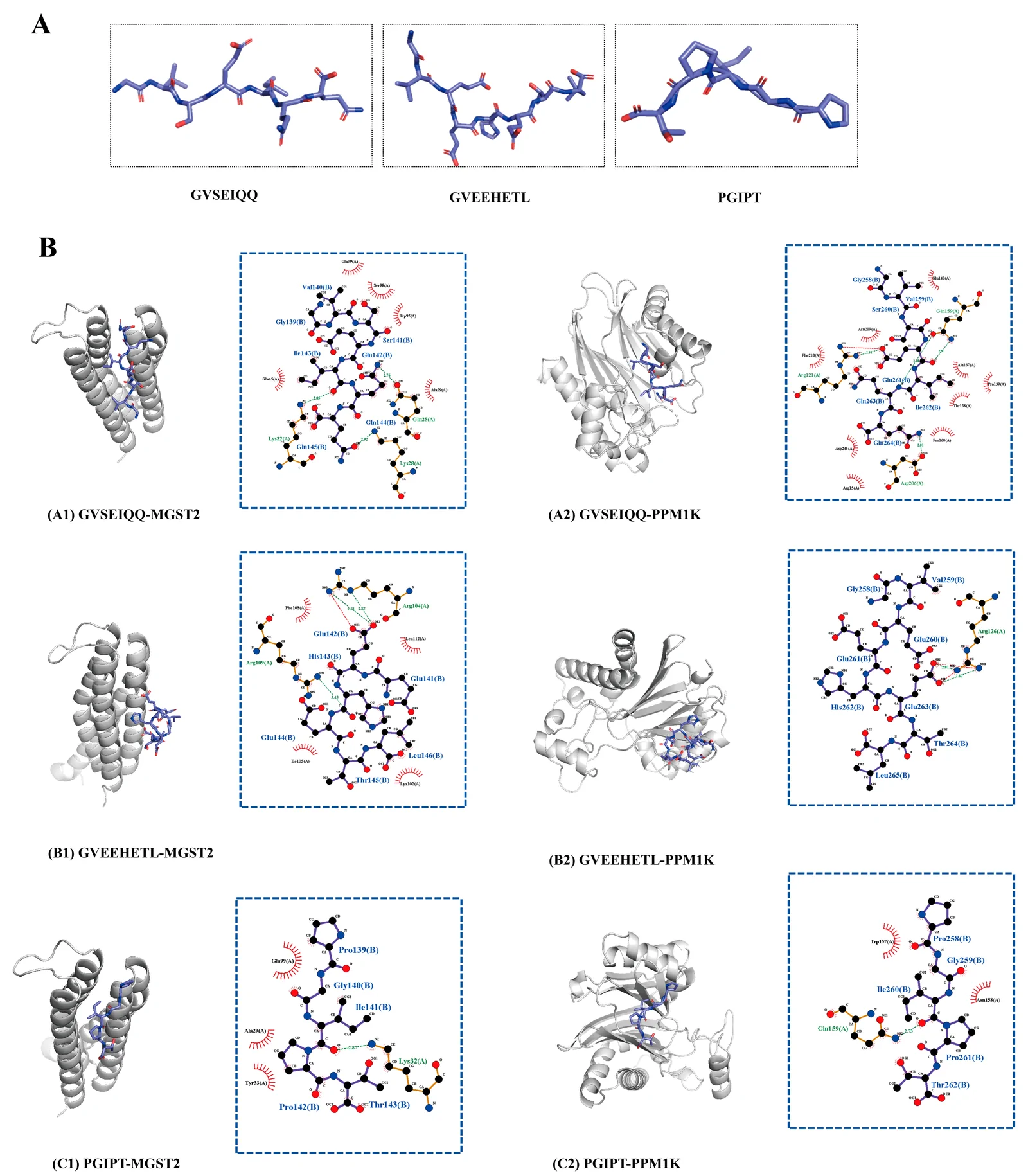
Molecular docking analysis of candidate peptides with MGST2 and PPM1K. (**A**) Three-dimensional structures of the candidate peptides. (**B**) (**A1**–**C1**) Predicted docking poses of the candidate peptides with MGST2; (**A2**–**C2**) Predicted docking poses of the candidate peptides with PPM1K.

**Figure 17:**
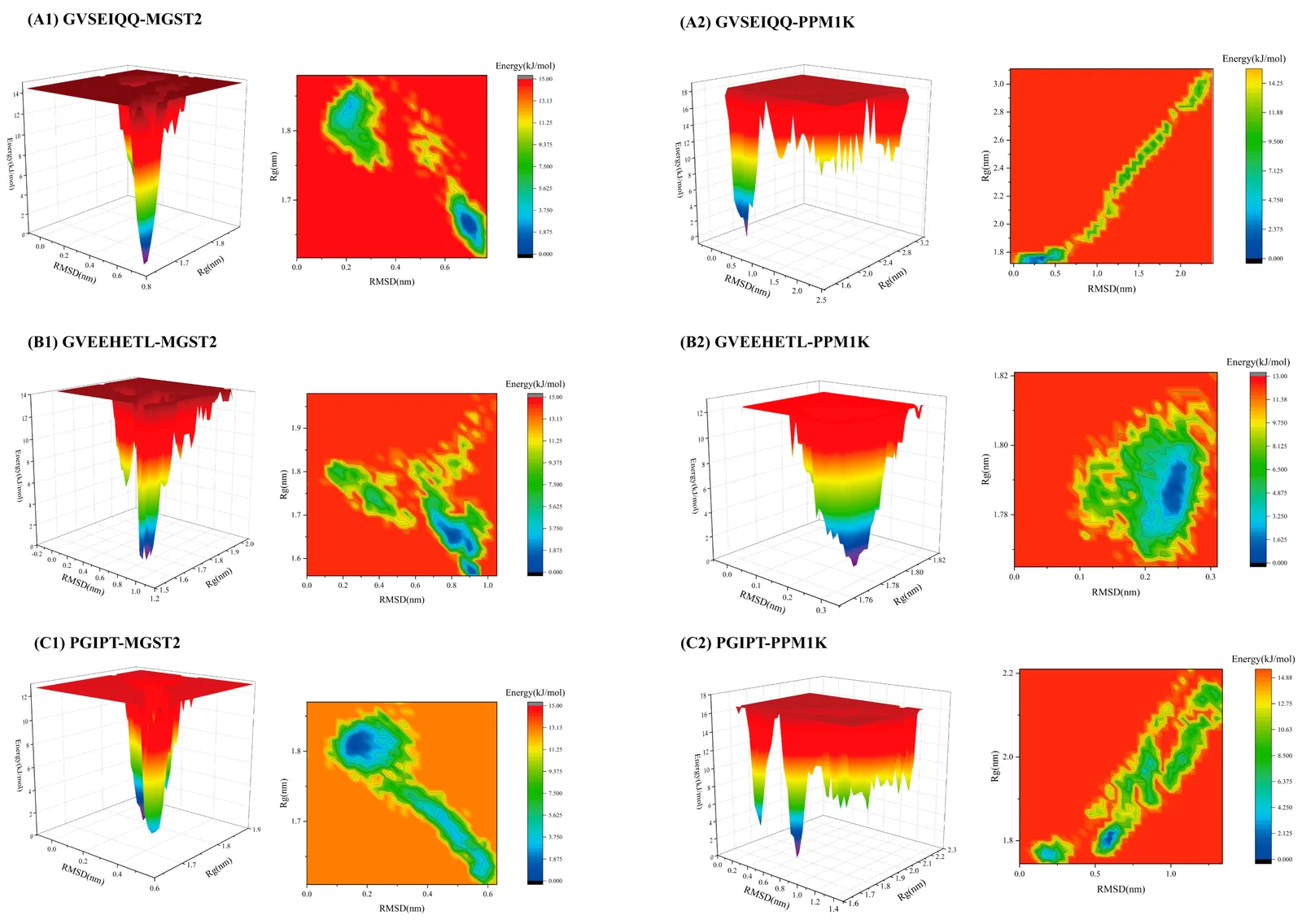
Free energy landscape analysis of peptide–MGST2 and peptide–PPM1K complexes. (**A1**–**C1**) Free energy landscape analysis of peptide–MGST2 complexes. (**A2**–**C2**) Free energy landscape analysis of the peptide–PPM1K complex.

To further investigate the potential interactions between the candidate peptides identified by serum peptidomics and the key target proteins MGST2 and PPM1K, molecular docking analysis of GVSEIQQ, GVEEHETL, and PGIPT with MGST2 and PPM1K was performed using HDOCK. The three-dimensional structures of the candidate peptides are shown in Figure 16A. The docking results showed that all three peptides could bind to the predicted active regions or potential binding pockets of MGST2 and PPM1K (Figure 16B), and their binding conformations were mainly maintained through non-covalent interactions, including hydrogen bonds and hydrophobic interactions. The HDOCK docking scores are presented in Table 7. For the MGST2 system, the docking scores of the three peptides were ranked as follows: MGST2–GVEEHETL (−136.67), MGST2–GVSEIQQ (−127.56), and MGST2–PGIPT (−117.82), suggesting that GVEEHETL may have relatively strong initial binding affinity for MGST2. For the PPM1K system, PPM1K–GVEEHETL showed the lowest docking score (−135.30), indicating its strong binding potential with PPM1K. To further evaluate the dynamic stability of the candidate peptides after binding to the target proteins, 100 ns molecular dynamics simulations were performed for the six peptide–protein complexes. RMSD, RMSF, number of hydrogen bonds, radius of gyration (Rg), solvent-accessible surface area (SASA), free energy landscape, and secondary structure changes were comprehensively analyzed. The results showed that all complexes formed stable conformations to varying degrees during the simulations, although their stability differed among different peptide–target protein combinations. In the MGST2 system (Figure S1), MGST2–GVSEIQQ and MGST2–GVEEHETL reached stability at approximately 40 ns and 60 ns, respectively, with relatively small fluctuations after equilibrium. In contrast, MGST2–PGIPT reached equilibrium later and exhibited larger fluctuations in the later stage, indicating relatively weaker stability. RMSF and hydrogen bond analyses showed that GVSEIQQ and GVEEHETL imposed stronger restrictions on MGST2 residue mobility and formed more hydrogen bonds during the simulation. Combined with the Rg, SASA, and free energy landscape results (Figure 17(A1–C1)), the MGST2–GVSEIQQ system showed lower Rg and SASA values and a more concentrated low-free-energy region, indicating that the complex formed by GVSEIQQ and MGST2 was more compact and conformationally stable. In the PPM1K system (Figure S2), PPM1K–GVEEHETL rapidly reached a stable state, with the final RMSD stabilizing at approximately 0.27 nm and showing only slight fluctuations. In contrast, PPM1K–GVSEIQQ and PPM1K–PGIPT showed higher RMSD values, suggesting more pronounced conformational adjustments. RMSF analysis showed that most PPM1K residues exhibited lower fluctuations after binding to GVEEHETL. Further integration of the Rg, SASA, and free energy landscape results (Figure 17(A2–C2)) revealed that PPM1K–GVEEHETL had the lowest and least fluctuating Rg value, a relatively low SASA value, and a more concentrated low-free-energy region, indicating that the complex formed by GVEEHETL and PPM1K was the most compact and stable. Secondary structure analysis showed that all complexes were mainly composed of stable structural elements, such as α-helices and β-sheets, whereas loops, turns, and bends exhibited only local fluctuations (Figure S3). These results indicate that peptide binding did not markedly disrupt the main secondary structural framework of MGST2 or PPM1K. Overall, GVSEIQQ may be a potential functional peptide with relatively high binding stability toward MGST2, whereas GVEEHETL may be the most stable candidate peptide for binding to PPM1K. These peptides may serve as key candidates for subsequent functional validation and mechanistic studies.

## 4. Discussion

*Periplaneta americana* residue (PAR) was partially defatted by ethanol soaking but retained a high crude protein content, a diverse amino acid profile, and certain bioactive constituents owing to the relatively limited extraction efficiency, highlighting its nutritional value and potential as an alternative protein feed ingredient. Biosafety evaluation showed that dietary supplementation with different levels of PAR caused no apparent histopathological abnormalities in major organs or significant changes in serum biochemical markers of liver and kidney function in mice, supporting its tolerability under the conditions tested. Based on our previous findings regarding reproductive performance in indigenous pig breeds and the aim of partially replacing soybean meal, a dietary PAR inclusion level of 3% was selected. We subsequently evaluated its effects on growth performance, serum oxidative stress markers, and ovarian multi-omics profiles in pigs. Supplementation with 3% PAR significantly increased average daily gain without significantly affecting average daily feed intake or the feed-to-gain ratio, suggesting improved growth performance without a detectable increase in feed intake. Hematoxylin and eosin staining revealed primordial, mature, and atretic follicles in both the CO and DL groups. Compared with the CO group, the DL group showed a slight numerical increase in primordial follicle number and no significant difference in mature follicle number. Notably, the number of atretic follicles was significantly lower in the DL group, suggesting that PAR supplementation may help reduce follicular atresia and support follicular development. Insect proteins are considered promising alternatives to conventional protein sources in pig diets because of their high protein content, favorable amino acid profiles, and potential immunomodulatory properties [4]. The growth response observed here may therefore reflect the combined contributions of residual proteins, small peptides, and other bioactive constituents in PAR. Furthermore, serum malondialdehyde (MDA) levels were significantly lower in the DL group, whereas the activities of major antioxidant enzymes and the levels of inflammatory cytokines remained unchanged. These findings are consistent with reduced lipid peroxidation, with no detectable changes in the measured systemic inflammatory markers. Previous studies have also reported that glycoproteins derived from *P. americana* promote tissue repair by attenuating inflammation, providing a rationale for the potential biological activity of constituents retained in PAR. However, the specific components responsible for the observed effects and their mechanisms of action remain to be established.

Ovarian development and follicular maturation are complex physiological processes that depend heavily on metabolic homeostasis and signaling regulation. Energy, amino acid, and lipid metabolism, together with redox balance and endocrine signaling, contribute to oocyte maturation, granulosa cell proliferation and differentiation, and follicular development. Warzych et al. [23] reported that glucose and fatty acid metabolism within the follicular microenvironment provides energy for oocyte maturation and influences developmental competence. Lipids serve not only as energy substrates but also as precursors for steroid hormones and signaling molecules, linking them closely to oocyte quality and follicular function [24]. In the present study, untargeted metabolomics revealed a clear separation between the ovarian metabolic profiles of the CO and DL groups, indicating that dietary PAR supplementation altered the overall metabolic state of porcine ovarian tissue. Insect protein is considered a promising alternative protein source, with growing interest in its nutritional value and effects on animal growth, digestion, immunity, and health [5]. As an insect-derived protein feed ingredient, PAR may therefore influence ovarian metabolism and signaling by supplying proteins, amino acids, small peptides, and other bioactive constituents. Canonical signaling pathways, including Wnt, Notch, and PI3K–Akt, participate in follicular development, granulosa cell proliferation and differentiation, and hormonal responses [2]. The metabolic alterations associated with PAR supplementation may thus reflect changes in nutrient availability and suggest potential remodeling of local ovarian energy metabolism, lipid metabolism, and endocrine signaling networks. Further amino acid metabolomic analysis revealed distinct ovarian amino acid profiles between the CO and DL groups. Taurine levels were elevated in the DL group, accompanied by enrichment of taurine and hypotaurine metabolism, sulfur metabolism, and glutathione metabolism. Taurine is a sulfur-containing amino acid derivative with cytoprotective properties that contributes to osmoregulation, mitochondrial function, and antioxidant defense. Previous research has demonstrated that taurine can attenuate oxidative stress, inflammation, and mitochondria-mediated apoptosis in porcine ovarian granulosa cells [25]. Cysteine is also an essential precursor for glutathione synthesis, and the glutathione system plays a key role in scavenging reactive oxygen species (ROS), maintaining cellular redox homeostasis, and preserving oocyte quality [26]. In addition, alterations in arginine and proline metabolism and argininosuccinate abundance suggest that the arginine–nitric oxide (NO) metabolic axis may contribute to the regulation of local ovarian blood flow, cell proliferation, and follicular development [27]. Elevated γ-aminobutyric acid levels and enrichment of related neuroactive pathways further suggest a metabolic response associated with neuroendocrine regulation in the ovaries of the DL group. Metabolite set enrichment analysis (MSEA) further linked the differential metabolites to estrogen, gonadotropin-releasing hormone (GnRH), prolactin, AMPK, mTOR, and insulin signaling pathways, as well as progesterone-mediated oocyte maturation and oocyte meiosis. AMPK and mTOR are key pathways involved in sensing cellular energy status and nutrient availability and are closely associated with follicular development, granulosa cell function, and oocyte maturation [28].

Transcriptomic, proteomic, and phosphoproteomic analyses provided support for the observed metabolic and physiological changes at multiple molecular levels. Transcriptomic analysis identified 874 differentially expressed genes, primarily involved in oxidative stress, nutrient metabolism, and cell fate regulation. Among the enriched pathways, ferroptosis provided a potential link between oxidative damage and follicular atresia. Ferroptosis is a form of regulated cell death characterized by iron-dependent lipid peroxidation and has been implicated in granulosa cell injury and follicular atresia [29,30,31]. Together with the lower serum MDA levels and reduced number of atretic follicles in the DL group, these ferroptosis-related transcriptional changes suggest that PAR supplementation may be associated with the regulation of lipid peroxidation and attenuation of follicular injury. Enrichment of the Wnt, Hippo, p53, and calcium signaling pathways further implicated processes involved in cell proliferation, differentiation, apoptosis, and follicular development [2,32], whereas enrichment of arachidonic acid and retinol metabolism suggested potential alterations in local lipid mediators and vitamin A metabolism [33,34]. These findings are consistent with the alterations in lipid metabolism and reproduction-related pathways identified by metabolomics, collectively suggesting an association between PAR supplementation and changes in ovarian metabolic and cell fate regulatory networks. However, pathway enrichment alone cannot establish whether a pathway is activated or inhibited, and reduced serum MDA levels do not directly demonstrate suppression of ferroptosis within the ovary. Further validation requires assessment of key ferroptosis-related proteins, including GPX4 and SLC7A11, together with measurements of Fe^2+^ accumulation and lipid reactive oxygen species in ovarian tissue.

Proteomic analysis identified 181 differentially expressed proteins, primarily associated with immune responses, phagocytic clearance, glutathione metabolism, and cell survival signaling. Follicular development, ovulation, and changes in the corpus luteum involve immune cell participation and tissue remodeling, with macrophage-mediated cellular clearance and local signaling playing important roles in maintaining the ovarian microenvironment [35,36]. Accordingly, enrichment of the phagosome, complement and coagulation cascades, Fcγ receptor-mediated phagocytosis, and NF-κB signaling pathways may reflect changes in local ovarian immune responses and tissue turnover following PAR supplementation. Enrichment of glutathione metabolism provided additional molecular evidence implicating antioxidant defense, consistent with the lower serum MDA levels and lipid peroxidation-related transcriptional changes. Enrichment of the PI3K–Akt and calcium signaling pathways further suggested potential effects on processes associated with cell survival and follicular development [37]. These changes in protein abundance link the metabolic and oxidative stress-related findings to the ovarian tissue microenvironment. However, they do not establish an improved inflammatory status or enhanced antioxidant function. Further validation requires measurements of local ovarian inflammatory mediators, the reduced-to-oxidized glutathione (GSH/GSSG) ratio, and the activities of key proteins. Phosphoproteomic analysis identified 960 differentially abundant phosphopeptides, primarily associated with cytoskeletal regulation and the cAMP, ErbB, and Hippo signaling pathways, providing insights into PAR-associated molecular changes at the post-translational level. cAMP signaling contributes to the regulation of oocyte meiosis, whereas the EGF/ErbB network mediates intrafollicular luteinizing hormone (LH) signaling and participates in cumulus expansion, oocyte maturation, and ovulation [38,39,40]. Changes in the corresponding phosphopeptides therefore suggest altered ovarian cellular responses to gonadotropins and local growth factors. Protein–protein interaction (PPI) clustering analysis further implicated MAPK, Ras, AMPK, and insulin signaling modules, suggesting that these changes may involve coordination between growth signaling and energy metabolism. The concurrent enrichment of actin cytoskeleton regulation and Hippo signaling also provided clues to a potential link between cellular structural remodeling and follicular growth [3,41]. However, differences in phosphopeptide abundance may reflect changes in either phosphorylation levels or the abundance of the corresponding proteins and therefore cannot be directly interpreted as changes in pathway activity. Further studies incorporating normalization to total protein abundance, measurement of phosphorylation at key sites, and functional perturbation are needed to establish the direction of these signaling changes and their functional significance for follicular development.

Serum peptidomics identified 1465 peptides, of which 58 were differentially abundant, suggesting that dietary PAR supplementation was associated with changes in the circulating peptide profile. Serum peptides can originate from dietary protein digestion, endogenous proteolysis, and peptide metabolism; thus, changes in their abundance may reflect systemic responses to nutritional intervention [42]. The precursor proteins of the differential peptides were primarily enriched in pathways related to cell adhesion, cytoskeletal regulation, phagosomes, and apoptosis, consistent with the changes involving tissue remodeling and immune responses observed in the ovarian omics analyses. Interactions between cells and the extracellular matrix are essential for follicular cell survival and follicular development [43]. However, functional enrichment of precursor proteins does not directly indicate the biological activities of the peptides themselves or establish their effects on the ovary. Three candidate peptides, GVSEIQQ, GVEEHETL, and PGIPT, were selected for further analysis. Computational toxicity and allergenicity analyses predicted all three peptides to be non-toxic and non-allergenic. AnOxPePred analysis suggested potential antioxidant activity for all three peptides, with GVEEHETL exhibiting the highest predicted free radical-scavenging score [44]. Together with the lower serum MDA levels in the DL group and the changes in ovarian glutathione metabolism and related processes, these findings provide a rationale for further investigation of the antioxidant effects of the candidate peptides. Nevertheless, they do not establish a causal relationship between these peptides and the improved ovarian phenotypes. Furthermore, whether these peptides originate directly from PAR remains unknown, and computational predictions cannot substitute for experimental safety and functional assessments. Further studies incorporating peptide source tracing, targeted quantification, in vitro antioxidant assays, and models of porcine ovarian granulosa cell injury are needed to validate their biological activities and clarify their availability in target tissues and potential mechanisms of action.

Integrated transcriptomic and proteomic analysis identified nine potential key targets, with changes in GPC6, CPN1, MGST2, and PPM1K supported by qPCR and Western blot validation. MGST2 participates in glutathione-dependent lipid hydroperoxide metabolism and leukotriene C4 synthesis, the latter of which has also been implicated in endoplasmic reticulum stress-induced reactive oxygen species accumulation and oxidative DNA damage [45]. In this study, altered MGST2 expression, enrichment of glutathione metabolism, reduced serum MDA levels, and predicted binding between the candidate peptides and MGST2 collectively provide a basis for further investigation of its involvement in PAR-associated redox regulation. However, MGST2 participates in both antioxidant metabolism and pro-oxidant responses; therefore, neither expression changes nor predicted binding alone can establish the direction of its effects. Further validation requires measurements of enzyme activity and lipid peroxidation, together with functional perturbation experiments. PPM1K was identified in both the integrated transcriptomic–proteomic and proteomic–phosphoproteomic analyses, highlighting it as an important candidate linking the different omics datasets. PPM1K is a mitochondrial protein phosphatase involved in branched-chain amino acid catabolism and, together with branched-chain α-ketoacid dehydrogenase kinase, contributes to the regulation of branched-chain amino acid and lipid metabolism [46]. Considering the alterations in the tricarboxylic acid (TCA) cycle and amino acid metabolism identified by metabolomics, along with the enrichment of AMPK signaling in the phosphoproteomic analysis, PPM1K may contribute to PAR-associated adjustments in ovarian energy metabolism. Immunofluorescence analysis revealed stronger PPM1K staining and significantly higher mean optical density in the DL group, consistent with the qPCR and Western blot results and supporting increased ovarian PPM1K expression following PAR supplementation. Nevertheless, increased expression does not necessarily indicate enhanced phosphatase activity, and differences in phosphopeptide abundance may also reflect changes in total protein abundance. Further studies should incorporate normalization to total protein abundance, assessment of key phosphorylation sites, and enzyme activity measurements, alongside knockdown or overexpression experiments, to establish its functional contribution. Other candidate molecules provided additional insights into the extracellular microenvironment and peptide metabolism. GPC6 participates in the regulation of cell-surface growth factor signaling and may be associated with the observed changes in cell adhesion, cytoskeletal organization, and tissue remodeling [47,48]. CPN1 contributes to inflammatory regulation by processing bioactive peptides, including complement-derived peptides and kinins [49], although a direct link to the differential serum peptides identified here has not been established. In addition, HRG, ITIH1, and GPRIN1 exhibited changes at both the protein and phosphopeptide abundance levels. The known functions of HRG and ITIH1 suggest that local immune regulation and extracellular matrix remodeling may contribute to PAR-associated responses.

Molecular docking and molecular dynamics simulations provided computational insights into potential interactions between the candidate peptides and MGST2 and PPM1K. For MGST2, GVEEHETL exhibited the most favorable docking score, followed by GVSEIQQ, and both peptides formed more hydrogen bonds and hydrophobic interactions than PGIPT. The 100 ns simulations indicated that the MGST2–GVSEIQQ and MGST2–GVEEHETL complexes were generally stable, whereas the MGST2–PGIPT system reached a relatively stable state later and exhibited greater fluctuations during the later stages. Among these complexes, MGST2–GVSEIQQ showed lower residue fluctuations, greater structural compactness, and a more concentrated distribution of low-energy conformations. The more favorable static docking score of GVEEHETL and greater dynamic stability of GVSEIQQ reflect the different aspects assessed by these methods: docking scores evaluate candidate binding poses, whereas molecular dynamics simulations examine conformational evolution and the persistence of interactions under defined simulation conditions. For PPM1K, GVEEHETL exhibited both the most favorable docking score and relatively high dynamic stability, providing consistent computational support for prioritizing this peptide–protein pair for experimental validation. Furthermore, the major secondary structural elements of all complexes remained largely stable, suggesting that the modeled binding poses were not accompanied by substantial disruption of the overall target protein structures. Functionally, MGST2 participates in glutathione-dependent lipid hydroperoxide metabolism and leukotriene C4 synthesis, linking it to both antioxidant metabolism and stress-associated pro-oxidant responses [50],. Together with the enrichment of glutathione metabolism and reduced serum MDA levels, these findings support GVSEIQQ and GVEEHETL as candidates for further investigation of MGST2-associated regulation of lipid peroxidation. However, predicted binding alone does not establish that these peptides exert antioxidant or ovarian protective effects through MGST2. PPM1K participates in mitochondrial branched-chain amino acid catabolism and related metabolic regulation, and its upregulation is consistent with the changes in amino acid and energy metabolism observed in this study. The predicted interaction between GVEEHETL and PPM1K therefore provides an additional avenue for investigating the potential involvement of candidate peptides in ovarian metabolic regulation. Nevertheless, docking scores and simulation stability cannot be directly equated with experimentally measured binding affinity, nor can they establish whether the candidate peptides activate or inhibit MGST2 or PPM1K. The 100 ns simulations are also constrained by the initial conformations, system setup, and extent of conformational sampling. Whether these peptides can reach the relevant intracellular compartments containing their target proteins in ovarian cells remains unclear. Future studies should prioritize validation of direct binding for GVSEIQQ–MGST2 and GVEEHETL–PPM1K, combined with enzyme activity assays, cellular uptake and localization analyses, and functional perturbation of the target proteins, to determine the direction of their effects and their functional consequences for redox homeostasis and energy metabolism in granulosa cells.

However, this study has several limitations. The pig feeding trial evaluated only a single dietary PAR inclusion level of 3%, precluding determination of the dose–response relationship and the optimal inclusion level. Although qPCR, Western blotting, and immunofluorescence supported the expression changes in the candidate targets, the absence of cell-based functional validation prevents the establishment of causal roles for MGST2 and PPM1K in PAR-associated ovarian phenotypic changes. Evidence for interactions between the candidate peptides and target proteins was derived exclusively from molecular docking and molecular dynamics simulations, without validation through direct binding or enzyme activity assays. Therefore, these results cannot establish experimental binding affinities or determine the direction of changes in target protein function. Although the serum peptide sequences were identified by peptidomics, their bioactivity, toxicity, and allergenicity were predicted solely using bioinformatic tools and should not be regarded as experimentally validated functional or safety outcomes. Furthermore, no animal supplementation or rescue experiments using synthetic candidate peptides were performed, preventing differentiation between the overall nutritional effects of PAR and the independent contributions of specific peptides. The in vivo stability, tissue distribution, and ability of these peptides to reach their targets also remain unclear. Although FDR correction was applied to the omics analyses, multi-omics associations do not establish causality, and the candidate molecules and proposed mechanisms require further confirmation in independent samples and functional experiments. Future studies should incorporate multiple dietary inclusion levels, cell-based functional interventions, and animal experiments using synthetic peptides to clarify peptide–target relationships and their functional contributions to ovarian physiology.

## 5. Conclusions

Dietary supplementation with PAR can partially replace soybean meal while improving growth performance, alleviating oxidative stress, and reducing follicular atresia in finishing pigs, potentially contributing to lower feed costs. However, further studies are needed to evaluate its effects on reproductive performance. PAR supplementation was associated with changes in molecular features related to ovarian redox regulation, energy metabolism, and protein phosphorylation, with MGST2 and PPM1K identified as candidate targets. The three candidate peptides, GVEEHETL, GVSEIQQ, and PGIPT, may play important regulatory roles in these processes. This study provides a theoretical basis for the application of PAR as a novel insect-derived protein feed resource and the valorization of medicinal residues from *Periplaneta americana*.

## Figures and Tables

**Table 1:** Ingredients and nutrient composition of the experimental diets.

Feed Ingredients (%)	CO	DL	Nutrient Composition	Nutrient Level
corn	81.25	82.37	Crude protein (%)	13.50
Soybean meal	12.50	8.4	Digestible energy (kcal/kg)	3402
			Lysine (%)	0.83
Expanded full-fat soybeans	3.00	3.00	Digestible lysine (%)	0.73
			Methionine (%)	0.25
soybean oil	0.62	0.46	Digestible methionine (%)	0.89
Calcium hydrogen phosphate	0.73	0.76		
limestone	0.83	0.84	Methionine (%)	0.26
table salt	0.20	0.20		
Premix	0.50	0.50	phosphorus (%)	0.41
Lysine	0.28	0.33		
Methionine	0.01	0.02		
Threonine	0.07	0.09		
Tryptophan	0.01	0.03		
defatted *Periplaneta americana*	0.00	3.00	digestible phosphorus (%)	0.24

**Table 2:** Nutrient composition of *Periplaneta americana* and defatted *Periplaneta americana* meal.

Nutrient Component (%)	Dried Whole Insect	Defatted Insect Meal
Crude protein	68.31	65.04
Crude fat	33.03	16.88
Crude fiber	6.12	7.84
Moisture	1.29	7.03
Ash	3.83	2.69
Calcium	0.20	0.21
Phosphorus	0.35	0.31

**Table 3:** Amino acid composition of *Periplaneta americana*, imported fish meal, and defatted *Periplaneta americana* meal.

Amino Acid (%)	Dried Whole Insect	Imported Fish Meal	Defatted Insect Meal
Aspartic acid	3.44	5.58	3.92
Threonine	1.66	2.64	1.88
Serine	1.73	2.35	1.94
Glutamic acid	4.84	8.64	5.34
Glycine	2.77	3.85	3.03
Alanine	3.34	3.96	3.70
Cysteine	0.36	0.79	0.41
Tyrosine	2.77	2.05	3.00
Phenylalanine	1.63	2.61	1.87
Lysine	2.38	4.85	2.71
Ammonia	1.71	0.83	1.23
Histidine	1.14	1.81	2.55
Arginine	2.31	3.37	2.50
Proline	2.26	2.35	3.19

**Table 4:** Effect of defatted *Periplaneta americana* on serum parameters of sows.

Project	CO	DL	*p*
MDA, nmol/mL	2.87 ± 0.08	2.60 ± 0.07	0.027
T-AOC, mmol/L	0.58 ± 0.01	0.56 ± 0.01	0.157
T-SOD, U/mL	51.87 ± 1.25	51.26 ± 0.64	0.671
GSH-Px, U/mL	782.78 ± 11.78	771.68 ± 6.46	0.428
CAT, U/mL	47.92 ± 3.59	48.32 ± 1.65	0.923
TNF-α, pg/mL	9.74 ± 0.90	9.32 ± 0.68	0.717
IL-1β, pg/mL	119.50 ± 4.44	115.01 ± 1.69	0.368
IL-6, pg/mL	103.87 ± 3.04	108.02 ± 2.74	0.335

**Table 5:** Significantly upregulated peptides in serum peptidomics.

Sequence	Length	Average Peptide Expression Level		Toxicity Prediction	Allergenicity Prediction	FRS Score
		DL	CO			
GVSEIQQ	7	694,333	321,666	Non-Toxin	no evidence	0.3489
GVEEHETL	8	219,333	–	Non-Toxin	no evidence	0.4759
PGIPT	5	1,990,000	–	Non-Toxin	no evidence	0.4064

“–“ indicates not detected.

**Table 6:** Gene-specific primers used in the present study.

Gene	Primer Sequence	Tm (°C)
GPC6	F: 5′-AGGGGGCAATGTCAATCTGG-3′	60
	R: 5′-CTTCAGCTTCCGGGGAACAT-3′	
CPN1	F: 5′-TGGTCGCAATCTTCCTCCAC-3′	60
	R: 5′-GCATTCGTTGTGCACCTTGT-3′	
MGST2	F: 5′-TTGCCACTTGTCTGGGTCTG-3′	60
	R: 5′-GGCCAAAACCCCCAGACTTA-3′	
PPM1K	F: 5′-GGAATAGTTTGGGGCAGCCT-3′	60
	R: 5′-GTGAGGACCAGGAAGCTGTC-3′	
β-actin	F: 5′-GGCACCACACCTTCTACAACGAG-3′	
	R: 5′-TCATCTTCTCACGGTTGGCTTTGG-3′	

**Table 7:** Molecular docking results of peptides with MGST2 and PPM1K.

Peptide	Protein	Hdock Score
GVSEIQQ	MGST2	−136.67
GVEEHETL		−127.56
PGIPT		−117.82
GVSEIQQ	PPM1K	−117.15
GVEEHETL		−135.30
PGIPT		−119.88

## Data Availability

The data presented in this study are available within the article and its Supplementary Materials. Additional data are available from the corresponding author upon reasonable request.

## Supplementary Materials

The following supporting information can be downloaded at: https://www.mdpi.com/article/10.3390/ani16182958/s1. Figure S1: Molecular dynamics simulation analysis of peptide–MGST2 complexes. (A) Root mean square deviation (RMSD). (B) Root mean square fluctuation (RMSF). (C) Radius of gyration (Rg). (D) Number of hydrogen bonds. (E) Solvent accessible surface area (SASA). (F) Box plot analysis; Figure S2: Molecular dynamics simulation analysis of peptide–PPM1K complexes. (A) Root mean square deviation (RMSD). (B) Root mean square fluctuation (RMSF). (C) Radius of gyration (Rg). (D) Number of hydrogen bonds. (E) Solvent accessible surface area (SASA). (F) Box plot analysis; Figure S3: Secondary structure analysis of peptide–MGST2 and peptide–PPM1K complexes. (A1–C1) Secondary structure analysis of peptide–MGST2 complexes. (A2–C2) Secondary structure analysis of the peptide–PPM1K complex.
