# Supplementary material for: Effects of Dietary Periplaneta americana Residue Supplementation on Porcine Ovaries and Screening Analysis of Functional Small Peptides
Source: Animals (Basel). 2026 Sep 20;16(18):2958. doi: 10.3390/ani16182958 (PMC13603663; doi:10.3390/ani16182958)
Supplement: Supplementary file 1 [file animals-16-02958-s001.zip › animals-4543226-supplementary.pdf]

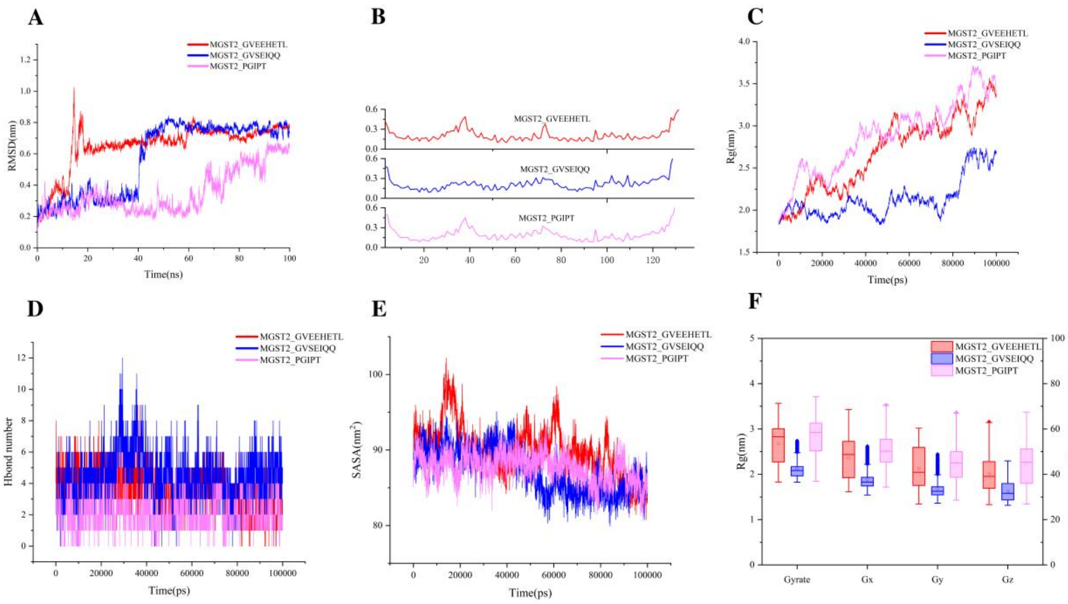

Figure S1 Molecular dynamics simulation analysis of peptide–MGST2 complexes.
(A) Root mean square deviation (RMSD).
(B) Root mean square fluctuation (RMSF).
(C) Radius of gyration (Rg).
(D) Number of hydrogen bonds.
(E) Solvent accessible surface area (SASA).
(F) Box plot analysis.

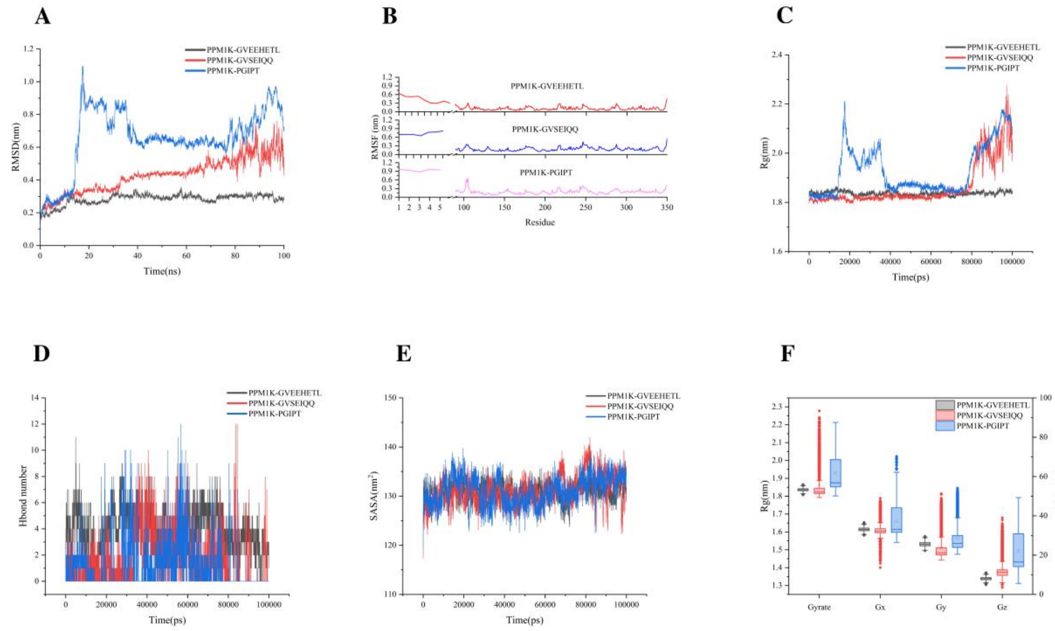

Figure S2 Molecular dynamics simulation analysis of peptide–PPM1K complexes.
(A) Root mean square deviation (RMSD).
(B) Root mean square fluctuation (RMSF).
(C) Radius of gyration (Rg).
(D) Number of hydrogen bonds.

(E) Solvent accessible surface area (SASA).
(F) Box plot analysis.

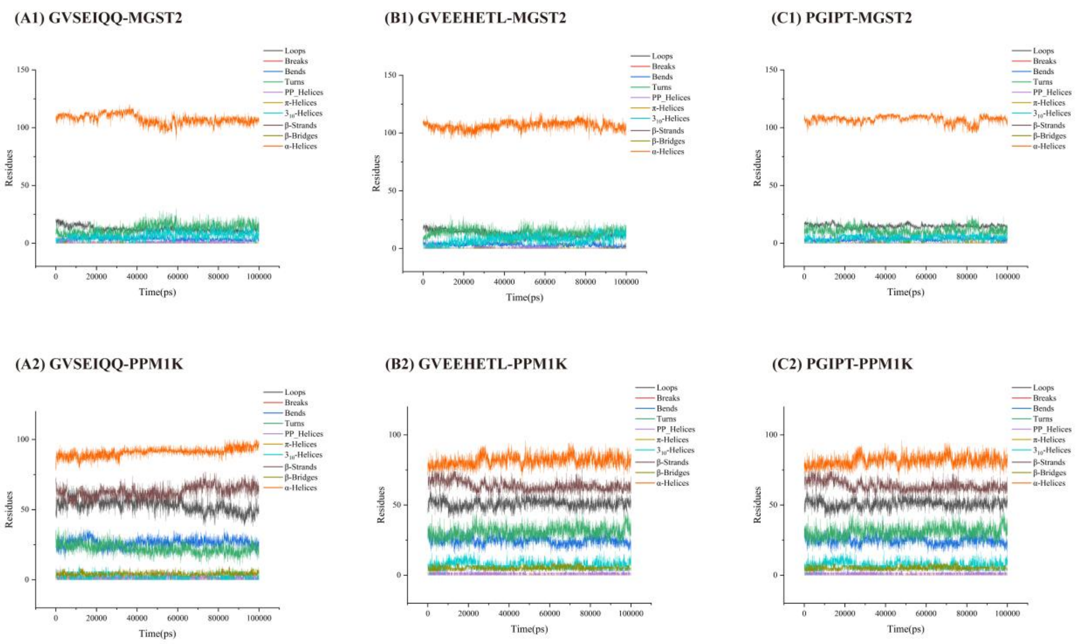

Figure S3 Secondary structure analysis of peptide–MGST2 and peptide–PPM1K complexes.

(A1-C1) Secondary structure analysis of peptide–MGST2 complexes.

(A2-C2) Secondary structure analysis of the peptide–PPM1K complex.
